# One Health Field Approach Applied to Leptospirosis: A Systematic Review and Meta-Analysis Across Humans, Animals and the Environment

**DOI:** 10.1093/ofid/ofae757

**Published:** 2024-12-30

**Authors:** Andrea Antoniolli, Hélène Guis, Mathieu Picardeau, Cyrille Goarant, Claude Flamand

**Affiliations:** Epidemiology and Public Health Unit, Institut Pasteur du Cambodge, Phnom Penh, Cambodia; Université Paris-Cité, Paris, France; Epidemiology and Public Health Unit, Institut Pasteur du Cambodge, Phnom Penh, Cambodia; CIRAD, UMR ASTRE, Phnom Penh, Cambodia; ASTRE, University of Montpellier, CIRAD, INRAE, Montpellier, France; Unité Biologie des Spirochètes, Institut Pasteur, Paris, France; Public Health Division, The Pacific Community, Nouméa, New Caledonia; Epidemiology and Public Health Unit, Institut Pasteur du Cambodge, Phnom Penh, Cambodia; Mathematical Modelling of Infectious Diseases Unit, Institut Pasteur, UMR2000, CNRS, Paris, France

**Keywords:** leptospirosis, one health, zoonosis, systematic review and meta-analysis

## Abstract

**Background:**

Leptospirosis is a neglected zoonosis transmitted through urine of infected hosts or contaminated environments. The transmission of bacteria between humans, animals, and the environment underscores the necessity of a One Health approach.

**Methods:**

We conducted a systematic review to identify significant findings and challenges in One Health research on leptospirosis, focusing on studies involving sampling in ≥2 of the 3 compartments: human, animal, and environment. We searched in PubMed, Web of Science, Medline, Scopus, and ScienceDirect from 1 January 1918 to 31 December 2022. We assessed risk of bias in studies using Joanna Briggs Institute tools and performed a meta-analysis to identify links between One Health compartments.

**Results:**

Of 1082 leptospirosis studies with sampling, 102 multicompartmental studies conducted between 1972 and 2022 were included: 70 human-Animal, 18 animal-environment, 4 human-environment, and 10 across all compartments. Various methodological weaknesses were identified, from study design to statistical analysis. Meta-regressions identified positive associations between human and animal seroprevalences, particularly with livestock and with wild nonrodent animals, and a link between the environmental positivity rate and domestic animal seroprevalence. Our analysis was constrained by the limited number of studies included and by the quality of protocols.

**Conclusions:**

This 50-year overview of One Health field approach to leptospirosis highlights the critical need for more robust, well-supported One Health research to clarify the transmission dynamics and identify risk factors of zoonoses.

## Teaser

Many studies exhibited methodological weaknesses from the design to the data analysis. Positive associations were found between human and animal, and animal and the environment. One Health is crucial to understand leptospirosis, but rigorous studies are needed.

Leptospirosis is a globally distributed bacterial zoonosis that affects mammals through leptospires penetration via wounds or mucous membranes. After colonization of the kidneys, the bacteria are excreted in urine [1]. Mammals may contract the infection directly from infected urine or through contact with environments contaminated by leptospires, where these bacteria can survive for several months [2].

The *Leptospira* genus comprises 69 species, classified into more than 250 serovars across more than 25 serogroups [3]. Due to its varied clinical manifestations, often characterized by influenzalike symptoms [3], leptospirosis is frequently misdiagnosed as other febrile illness like dengue or malaria, leading to its significant underestimation. With estimates of >1 million cases and about 60 000 deaths annually, predominantly in tropical regions [4], leptospirosis remains a neglected tropical disease [5, 6]. The diversity of serovars complicates serological diagnosis due to the low cross-reactivity of serovar-specific antibodies. The microscopic agglutination test (MAT), despite being the gold standard for serological diagnosis, has several limitations [7], contributing to the disease's neglect.

Host specificity of serovars varies widely, some serovars infect a broad range of hosts while others are host specific. Host roles in transmission also differ. Susceptible hosts might experience severe illness or death, excreting leptospires only during the acute phase, whereas maintenance hosts may show mild or no symptoms but continuously excrete leptospires for months or their entire lifespan, thus acting as reservoirs [3]. This enduring excretion is facilitated by coadaptation between host and serovar, like Icterohaemorrhagiae and rats or Hardjo and cattle [1].

Despite ongoing research, significant uncertainties about leptospires transmission remain, especially concerning regional and contextual variations. The environment's role is particularly unclear even though many outbreaks occur after heavy rainfalls or floods [2, 3]. The environment could serve as an intermediary between reservoirs and humans, potentially acting as a vector by dispersing leptospires. Heavy rainfalls might cause contaminated surface soil to slip into watercourses, carrying leptospires to humans. In addition, floods can transport leptospires, leading to new exposures [8]. This issue may grow with climate change and its effects on flooding [9] and rainfall [10]. Moreover, the role in disease transmission of dogs, often in close contact with humans [3], is not well understood.

Addressing uncertainties requires a One Health approach that integrates human, animal and environmental health disciplines [11] to clarify each compartment's role in pathogen dissemination, thereby enhancing our understanding of transmission dynamics and informing mitigation strategies.

This systematic review aims to provide a comprehensive overview of One Health field approaches to leptospirosis, identifying their strengths, challenges, and research quality. It also aims to guide future robust and integrated studies. Furthermore, through meta-analysis, this review seeks to identify factors linked to *Leptospira* presence or seroprevalence across the 3 compartments and explore intercompartment connections to elucidate transmission pathways.

## METHODS

### Study Design

We conducted a systematic review and meta-analysis following the Preferred Reporting Items of Systematic Review and Meta-Analysis (PRISMA) guidelines [12]. The protocol was registered on PROSPERO (CRD42023394574) through the National Institute for Health and Care Research [13] on 21 March 2023.

### Study Eligibility

This review focused on field studies incorporating the One Health concept, specifically those involving ≥2 of the 3 One Health compartments: human, animal, and environmental. Eligible studies needed to report *Leptospira* presence or exposure across these compartments, with clear descriptions of sample sizes. We excluded single-compartment studies, reviews, case reports, editorial comments, and studies without original field data. No language restrictions were applied.

### Data Source and Search Strategy

We searched databases including Scopus, Web of Science, PubMed, ScienceDirect and Medline from 1 January 1918 to 31 December 2022. The search strategy for Web of Science is detailed in Supplementary Material 1 and Supplementary Table 1. We excluded unpublished manuscripts and translated non-English and non-French studies using digital tools, verified by native speakers when necessary.

### Data Extraction and Quality Assessment

Titles and abstracts were initially screened, followed by full-text evaluations. Two reviewers resolved disagreements through discussion, involving a third arbitrator if necessary. Data were independently extracted by 2 reviewers into a structured Microsoft Excel form, and the risk of bias was assessed. Extracted information included study design, population, sample types, laboratory tests, data analysis, study limitations, and conclusions. We also collected data on prevalence, type of laboratory test, number positive, and total tested for each sample type. Study designs were classified into 6 categories, including a “mixed” category for diverse designs. Populations were categorized into 5 groups, also including a mixed category for various types: general population, febrile population, exposure to positive cases, and population at risk due to a given practice or lifestyle. The sample size calculation was complete if it covered human and animal species and partial if it omitted any of these. Randomization was assessed similarly, with wildlife trapping considered inherently random and environmental sampling was considered unsuitable for randomization, given the difficulty of achieving representativeness.

The quality of each study was assessed using the Joanna Briggs Institute (JBI) critical appraisal tools (presented in Supplementary Figures 1 and 2) tailored to the study design [14, 15]. Populations were categorized into 4 groups: humans, domestic animals, wild animals, and the environment. Each group's protocol quality was evaluated independently, with inappropriate questions omitted from the JBI tool to ensure standardized scoring. Scores were normalized to 1 for comparability across groups.

### Data Synthesis and Statistical Analysis

Data analyses were conducted using R software, version 4.3.1 [16]. Meta-analyses were performed via generalized linear random-effects models to estimate subgroup-aggregated proportions using the rma.glmm function from the metafor R package [17]. These models accounted for variability among studies due to heterogeneity [18]. Pooled proportions of individuals testing positive for bacteria or antibodies were expressed as percentages with 95% confidence intervals (CIs). Data were logit transformed for modeling and subsequently inverse-logit transformed for generating estimates and forest plots [19]. Heterogeneity was quantified using the *I*² statistic [19]. For the meta-analysis, all studies were included, regardless of their JBI score. For animal data, each species from a study was considered separately, and for environmental data, each sample type (water or soil) was treated individually. Consequently, multiple proportions from the same study could be included in the meta-analysis, appearing separately for different species or sample types. For environmental data, the positivity rate was calculated by combining findings of polymerase chain reaction (PCR) and culture. Subgroups were established based on study population type, geographic region, animal type, species or subfamilies, and environmental sample type. Subgroups of species were considered if included in ≥10 studies.

Generalized linear mixed-effects models were used to assess the impact of moderator variables on outcomes, incorporating fixed effects of variables like study population type, geographic region, country, animal type, species and observed proportion rates. Adjustments were based on study population type: febrile, general, at risk, exposed, or mixed. There were 2 conditions for testing an association by explaining one positivity rate by another. First, we fixed the minimum sample size at 10 studies: ≥10 studies had to measure the 2 positivity rates to be tested. Second, a positivity rate was used as an explanatory variable only if it was measured in ≥10 individuals.

## RESULTS

### Eligible Studies

**Figure 1.**
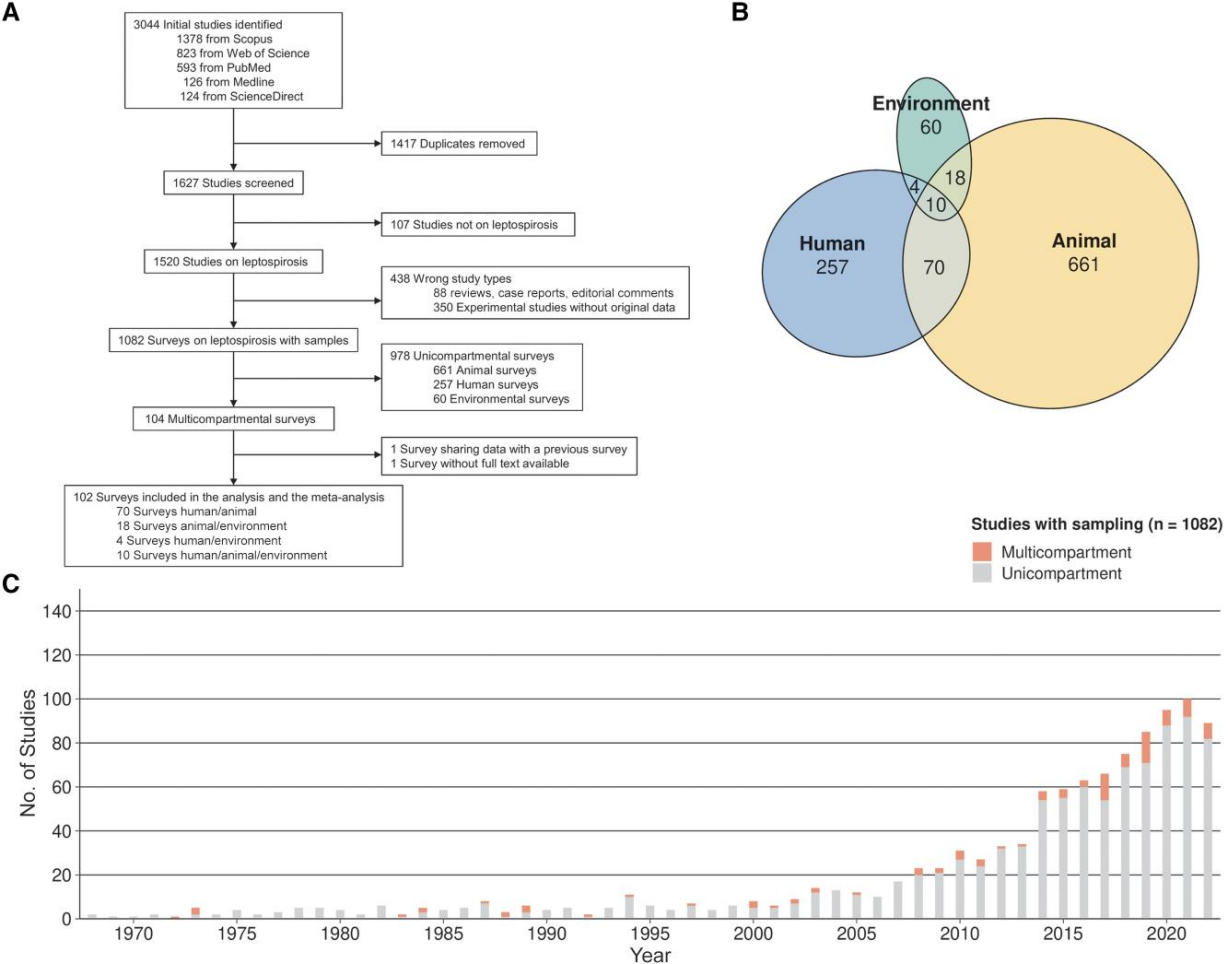
Implementation of the One Health approach. *A*, Flowchart of study selection. *B*, Venn diagram showing the numbers of studies with sampling, according to the compartment(s) investigated. *C*, Numbers of unicompartmental and multicompartmental studies published per year.

We initially identified 3044 studies. After removing 1417 duplicates and screening 1627 studies, we included 102 in the review, covering the period from 1972 to 2022 (Figure 1*A*). We excluded 978 unicompartmental surveys and 1 study [20] with redundant data from a previous article [21]. Another study was excluded due to the unavailability of the full text [22].

Figure 1 *B* displays a Venn diagram illustrating intersections among the 102 One Health studies included. The environmental compartment was the least represented in unicompartmental studies (n = 60 [6.1%]) and appeared in only 30.7% of multicompartmental studies. In contrast, animals and humans were included in 94.2% and 82.4% of studies, respectively. Only 10 studies (9.8%) investigated all 3 compartments. The use of multicompartmental approaches studying leptospirosis significantly increased after the year 2000 (Figure 1*C*), but overall such approaches were used in only 9.6% of published studies.

### Database Description

**Figure 2.**
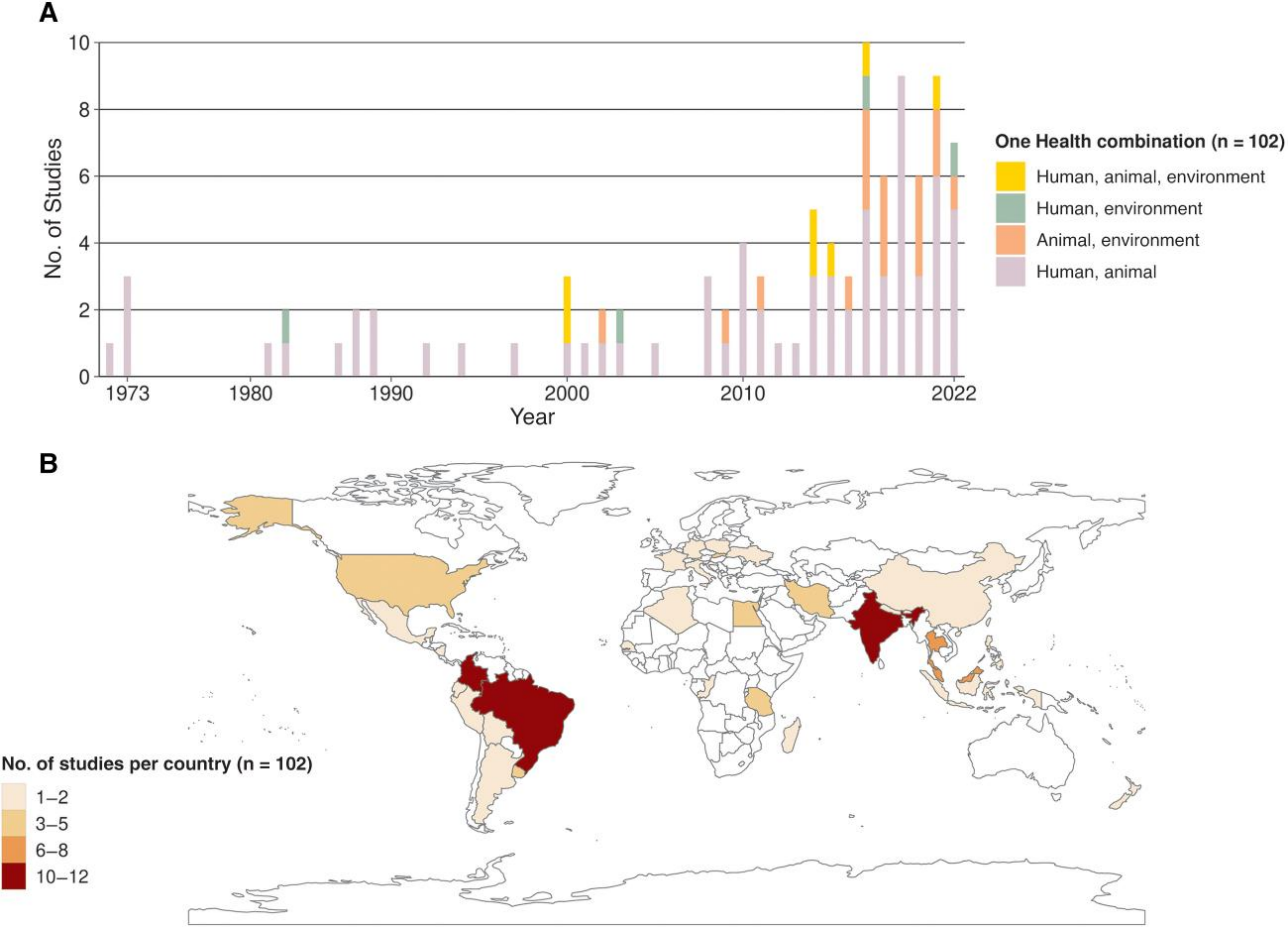
Spatiotemporal distribution of included studies. *A*, Number of studies included per year, showing compartments of the One Health concept investigated. *B*, Spatial distribution of the numbers of studies by country.

The investigation of the environment has progressed more gradually compared with the other 2 compartments (Figure 2*A*). Before 2000, only 6% of studies (1 of 15) involved the environment, compared with 36% (31 of 87) after 2000. Most studies were conducted in South America (n = 36 [35.3%]) or Asia (n = 34 [33.3%]) (Figure 2*B*). Supplementary Table 2 provides the detailed geographic distribution.

Table 1 describes the characteristics of included studies. Among the 98 animal studies, 44% focused on domestic animals, 22% on wild animals and 34% on both. Of the 76 studies investigating domestic animals, 33% investigated livestock, 26% pets, and 41% both. Among the 32 environmental studies, 22 (69%) investigated water, 9 (28%) both water and soil, and 1 (3%) examined only the air. Environmental samples were mainly from nearby farms (38%) or houses (28%), with 34% involving domestic water and 34% waterways.

**Table 1.** Characteristics of One Health Studies on Leptospirosis Included in the Current Review

Study Authors (Year)	Type	Date	Country	Design	Population	Test	Positivity Rate
Maronpot and Barsoum (1972) [23]	H, A	1968–1971	Egypt	Prevalence survey	H: mix; A: gen	MAT	H 52/513, cows 209/424, pigs 70/130, goats 135/195, sheep 57/330, horses 11/31, camels 29/50
Nelson et al (1973) [24]	H, A	1964	USA	Cluster investigation	H: feb; A: exp	MAT	H 61/245, cows 26/305
						CAR	Cows 9/43
Tsai et al (1973) [25]	H, A	1968	Taiwan	Prevalence survey	H: gen A: gen	MAT	H 25/167, dogs 0/2, cows 18/57, pigs 0/64, goats 2/32, civets 1/2, rodents 3/47
						Culture	Civets 1/2, rodents 7/59
Limpias and Marcus (1973) [26]	H, A	…	Bolivia	Prevalence survey	H: feb A: mix	MAT	H 7/142, dogs 1/17, cows 372/520, pigs 7/102, goats 4/53, sheep 1/61, horses 76/101, monkeys 1/1, bats 0/9, rabbits 0/1, snakes 0/1, boars 1/1, deer 1/1, rodents 0/3
Ratnam et al (1983) [27]	H, A		India	Cluster investigation	H: mix A: feb	MAT	H 35/75, cows 27/40
						Culture	Cows 0/9
Prokopcaková and Pospisil (1984) [28]	H, A	…	Slovakia	Prevalence survey	H: gen; A: gen	ALR	H 28/876, cows 13/168, rodents 20/243
Gelosa and Manera (1984) [29]	H, E	1980–1983	Italia	Laboratory monitoring	H: feb	MAT	H 34/168
						Culture	Water 0/40
Sebek et al (1987) [30]	H, A	1976–1982	Iran	Prevalence survey	H: feb; A: gen	MAT	H 80/2448, cows 2/4, pigs 15/89, goats 6/65, sheep 9/328
Heisey et al (1988) [31]	H, A	1983–1984	Thailand	Prevalence survey	H: feb; A: feb	MAT/Culture	H 33/110
						MAT	Dogs 56/293, cats 0/8, cows 54/204, pigs 3/17, rodents 84/174
						Culture	Cows NA/150, rodents 23/75
Everard et al (1988) [32]	H, A	1982–1984	Belize	Prevalence survey	H: mix A: mix	MAT	H 99/451, dogs 5/7, cows 136/155, pigs 32/71, goats and sheep 88/162
Sebek et al (1989) [33]	H, A	1987	Egypt	Prevalence survey	H: gen; A: gen	MAT	H 17/196, dogs 0/1, cows 0/1, pigs 4/28, goats 1/67, horses 2/12, rodent 36/65
Sebek et al (1989) [34]	H, A	1983	Cape Verde	Prevalence survey	H: mix; A: gen	MAT	H 44/611, dogs 0/89, cows 3/150, pigs 0/316, goats 34/640, sheep 0/39, horses 3/64, rodents 0/211
Venkataraman and Nedunchelliyan (1992) [35]	H, A	1988	India	Cluster investigation	H: feb; A: mix	MAT	H 48/95, dogs 20/94, bandicoots 10/24, rodents 8/64
						DFM	H 10/48+,^a^ dogs 8/20+, bandicoots 8/24+, rodents 5/32
						Culture	H 1/10+, dogs 1/8+
Prokopcáková et al (1994) [36]	H, A	1991–1993	Slovakia	Prevalence survey	H: risk; A: gen	MAT	H 56/1740, rodents 99/1038, shrews 1/68
Machang’u et al (1997) [37]	H, A		Tanzania	Prevalence survey	H: risk; A: gen	MAT	H 1/375, dogs 80/208, cows 28/374, rodents 10/537
						Culture	Cows 7/1021
Campagnolo et al (2000) [38]	H, A, E	1998	USA	Cluster investigation	H: risk; A: feb	IgMELISA	H 9/17
						MAT	Pigs 97/302
						Culture	Pigs 4/6, water 0/8
Ochoa et al (2000) [39]	H, A	1997–1998	Colombia	Prevalence survey	H: risk; A: gen	MAT	H 15/67, cows 106/174, pigs 60/278
Vanasco et al (2000) [40]	H, A, E	1998	Argentina	Cluster investigation	H: mix; A: exp	MAT	H 12/32, dogs 6/8
						DFM	Water 8 spirochete+/8
						Culture	Water 8 spirochete+/8
Ralaiarijaona et al (2001) [41]	H, A	2000	Madagascar	Prevalence survey	H: risk; A: gen	MAT	H 1/105
						PCR	Cows 0/50, pigs 0/13, rodents 0/115
León et al (2002) [42]	A, E	1996–1998	Colombia	Prevalence survey	A: feb	MAT	Pigs 0/68
						DFM	Water 91/339
						Culture	Water 38/311
Natarajaseenivasan et al (2002) [43]	H, A	2000	India	Mixed	H: mix; A: gen	IgGELISA	H 241/268
						MAT	H 231/338, dogs 2/4, cats 6/9, cows 18/34, rodents 12/23
Ramakrishnan et al (2003) [44]	H, E	2001	India	Cluster investigation	H: exp	MAT	H 20/64
						Culture	Water 1/1
						PCR	Water 1/1
Cerri et al (2003) [45]	H, A	1995–2001	Italia	Laboratory monitoring	H: feb; A: feb	MAT	H 14/250, dogs 278/4369, cows 7/644, pigs 123/1299, sheep 132/1088, horses 107/938, boars 11/459, deer 0/567, wolves 0/4, marmots 0/120, rodents 0/4
Ren et al (2005) [46]	H, A	1998–2003	China	Longitudinal monitoring	H: gen; A: gen	MAT	H 57/1777, pigs 10/232
						Culture	Dogs 0/30, pigs 1/524, rodents 16/123
Kuriakose et al (2008) [47]	H, A	1993–1997	India	Longitudinal monitoring	H: gen; A: gen	MAT	H 38/376, bandicoot 4/9, shrews 0/5, rodents 2/40
						DFM	Bandicoots 2/4, rodents 1/2
						Culture	Bandicoots 0/2, shrews 0/2, rodents 1/6
Langoni et al (2008) [48]	H, A	2005	Brazil	Prevalence survey	H: risk; A: gen	MAT	H 8/34, cows 46/140, rodents 0/50
						Culture	Cows 0/140, rodents 0/50
						PCR	Rodents 0/50
Habuš et al (2008) [49]	H, A	2007	Croatia	Laboratory monitoring	H: feb; A: mix	MAT	H 24/113, dogs 2/20, cows 295/9867, pigs 1397/15524, goats 24/1639, sheep 46/16278, horses 196/1212, foxes 36/70, wild animals (undefined) 0/100
Zhou et al (2009) [50]	H, A	2002	China	Prevalence survey	H: gen; A: gen	MAT	H 444/772
						Culture	Cows 11/225, rodents 22/726
Aviat et al (2009) [51]	A, E	2001–2004	France	Prevalence survey	A: exp	MAT	Rodents 288/649
						PCR	Rodents 41/516, water 6/151
Silva et al (2010) [52]	H, A	2006	Brazil	Prevalence survey	H: risk; A: gen	MAT	H 0/15, Snake 47/110, Fish 2/25, Bird 34/143, Wild undefined 11/49
Zakeri et al (2010) [53]	H, A	2005–2007	Iran	Prevalence survey	H: feb; A: gen	PCR	H 98/369, dogs 33/150, sheep 13/175
Romero et al (2011) [54]	H, A	2007	Colombia	Prevalence survey	H: gen; A: gen	MAT	H 51/850, dogs 182/850
Bermúdez et al (2010) [55]	H, A	…	Colombia	Prevalence survey	H: gen; A: gen	MAT	H 10/46, dogs 41/61
Cárdenas-Marrufo et al (2011) [56]	A, E	2004–2005	Mexico	Prevalence survey	A: gen	MAT	Dogs 22/61, cows 97/212, pigs 26/203
						PCR	Water 0/68
De Castro et al (2011) [57]	H, A	2007–2009	Brazil	Mixed	H: feb; A: gen	MAT	H 7/97, dogs 76/268
Romero et al (2011) [54]	H, A	2009–2010	Colombia	Prevalence survey	H: risk; A: gen	MAT	H 5/20, monkeys 15/65
Fonzar and Langoni (2012) [58]	H, A	2006–2008	Brazil	Prevalence survey	H: feb; A: gen	MAT	H 5/25, dogs 41/335
Romero-Vivas et al (2013) [59]	H, A	2007–2009	Colombia	Cluster investigation	H: feb; A: exp	MAT	H 16/128, dogs 19/83, rodents 13/69
						Culture	H 0/10, dogs 0/54, rodents 1/69
						PCR	H 1/10 PCR, dogs 2/4 (2+), rodents 2/16
Calderón et al (2014) [60]	H, A, E	2009–2011	Colombia	Prevalence survey	H: risk; A: gen	MAT	H 47/62, dogs 19/54, pigs 214/383, rodents 0/39
						Culture	Dogs 2/54, pigs 3/171, rodents 1/39, water 9/57
						PCR	Dogs 2/2+, pigs 3/3+, water 2/9+
Soman et al (2014) [61]	H, A		India	Prevalence survey	H: feb; A: mix	MAT	H 84/154, dogs 44/121, wild animals (undefined) 9/42
						Culture	H 1/154, dogs 1/121, bandicoots 3/11, rodents 2/24
Vimala et al (2014) [62]	H, A	2009–2010	India	Prevalence survey	H: feb; A: gen	MAT	H 10/129, rodents 9/24
Silva et al (2014) [63]	H, A	2013	Brazil	Prevalence survey	H: risk; A: gen	MAT	H 2/10, dogs 6/12, sheep 7/34, horses 6/10, rodents, 1/1, feral cats 0/1, foxes 1/2, tatous 0/16
Assenga et al (2015) [64]	H, A	2012–2013	Tanzania	Prevalence survey	H: gen; A: gen	MAT	H 80/267, cows 346/1141, goats 22/248, lions 1/2, zebras 0/2, shrews 1/11, rodent 42/207
Samir et al (2015) [65]	H, A, E		Egypt	Cluster investigation	H: exp; A: mix	MAT	H 87/175, dogs 98/168, cows 239/651, sheep 45/99, horses 2/40, camels 0/22, rodents 205/270
						Culture	H 0/175, dogs 19/168, cows 7/651, sheep 0/99, horses 0/40, camels 0/22, rodents 17/270
						PCR	H 0/175, dogs 65/168, cows 7/651, horses 0/40, sheep 0/99, camels 0/22, rodents 65/270, water 10/45
Da Silva et al (2015) [66]	H, A	2012	Brazil	Prevalence survey	H: gen; A: gen	MAT	H 11/28, dogs 7/13, cows 6/17, goats 16/37, sheep 16/41, horses 30/57, foxes 6/11, opossums 1/1, tatous 4/4, monkeys 3/4, coatis 2/3, rodents 1/1
Lugo-Chávez et al (2015) [67]	H, A	2012	Mexico	Cluster investigation	H: exp; A: gen	MAT	H 22/36, dogs 19/29
Barragan et al (2016) [68]	H, A	2013–2015	Ecuador	Prevalence survey	H: feb; A: gen	PCR	H 100/680, cows 59/165, pigs 27/128, rodents 3/101
Cibulski and Wollanke (2016) [69]	A, E	…	Germany and Luxembourg	Prevalence survey	A: gen	PCR	Shrews 3/67, Mole 1/1, rodents 38/226, water 9/87
Parveen et al (2016) [70]	H, A		India	Prevalence survey	H: risk; A: gen	MAT	H 94/244, dogs 4/15, cows 39/86, goats 7/29, rodents 9/23
						Culture	Rodents 2/23
Habus et al (2017) [71]	H, A	2009–2014	Croatia	Laboratory monitoring	H: feb; A: mix	MAT	H 395/1917, dogs 85/364, cows 3251/22 669, pigs 2016/18 163, goats and sheep 376/41 752, horses 5595/41 538
Chadsuthi et al (2017) [72]	H, A	2010–2015	Thailand	Laboratory monitoring	H: feb; A: feb	MAT	H 471/1990, cows 1133/4080, pigs 356/3138
Pui et al (2017) [73]	A, E	2014–2015	Malaysia	Prevalence survey	A: gen	PCR	Rodents 23/107, water 13/324, soil 46/292
Kurilung et al (2017) [74]	H, A, E	2013–2016	Thailand	Prevalence survey	H: gen; A: gen	Culture	H 0/37, dogs 4/58, cows 1/131, pigs 6/152, goats 0/1, horses 0/1, water 0/14
						PCR	H 1/37, dogs 6/58, cows 16/131, pigs 12/152, goats 1/1, water 3/14
Ensuncho-Hoyos et al (2017) [75]	H, A, E		Colombia	Prevalence survey	H: risk; A: gen	MAT	H 14/20, dogs 5/11, cows 242/325, water 0/39
						Culture	Cows 3/78
						PCR	Cows 3/3+, water 1/39
Jorge et al (2017) [76]	H, A	2003–2007	Brazil	Laboratory monitoring	H: feb; A: feb	MAT	H NA/997, dogs NA/1176, cows NA/1484, horses NA/240
Meny et al (2017) [77]	H, E	2010–2016	Uruguay	Cluster investigation	H: feb	MAT	H 5/302
						Culture	H 8/302, water 7/36
						PCR	H 8/8+, water 6/7+
Pui et al (2017) [78]	A, E	2014–2015	Malaysia	Prevalence survey	A: gen	PCR	Rodents 1/31, water 17/210, soil 8/210
Sanhueza et al (2017) [79]	H, A	2009–2013	New Zealand	Prevalence survey	H: risk A: gen	MAT	H 12/178, cows 717/1374, sheep 939/2178, Deer 72/1133
Grevemeyer et al (2017) [80]	A, E	…	Saint Kitts and Nevis	Prevalence survey	A: gen	PCR	Horses 22/124, water 0/2
Biscornet et al (2017) [81]	H, A	2013–2015	Seychelles	Prevalence survey	H: feb; A: gen	IgMELISA	H 18/223
						MAT	H 19/223
						PCR	H 32/223, dogs 1/24, cats 1/12, rodents 57/739
Chávez et al (2018) [82]	A, E	2014–2016	Nicaragua	Cluster investigation	A: exp	MAT	Dogs NA/159, cats NA/1, cows NA/36, pigs NA/60, horses NA/7
						Culture	Dogs NA/75, cows NA/15, pigs NA/22, water 61/129, soil 14/69
Shrestha et al (2018) [83]	H, A	2013	Nepal	Cluster investigation	H: feb; A: exp	MAT	H 13/239, dogs 9/20, cows 60/155, goats 31/181, rodents 3/14
Zala et al (2018) [84]	A, E	2016–2017	India	Longitudinal monitoring	A: gen	PCR	Dogs 2/30, cows 20/121, goats 1/40, soil 8/60, water 80/216
Cortez et al (2018) [85]	A, E	2014–2015	Peru	Longitudinal monitoring	A: gen	Culture	Water 1/64
						PCR	Rodents 23/97, water 23/64, soil 21/25
Tabo et al (2018) [86]	H, A	2015	Philippines	Prevalence survey	H: risk; A: gen	MAT	H 7/46, cows 3/9, pigs 37/69
Ukhovskyi et al (2018) [87]	H, A	2009–2016	Ukraine	Laboratory monitoring	H: feb; A: feb	MAT	H 3012/24 990, cats 52 310/1 238 876, pigs 31 181/989 659, horses 6734/70 674
Markovych et al (2019) [88]	H, A	2005–2015	Ukraine	Mixed	H: feb; A: gen	MAT	H 401/2079, rodents 276/2820
Takhampunya et al (2019) [89]	H, A	2014–2018	Thailand	Prevalence survey	H: feb; A: gen	PCR	H 3 pools/23 pools (200), rodents 3pools/64pools (309)
Salmon-Mulanovich (2019) [90]	H, A	2011–2014	Peru	Prevalence survey	H: gen; A: gen	MAT	H 229/2165, dogs 44/53, cats 2/10, Poultry 30/37, rodents 2/30
Jittimanee and Wongbutdee (2019) [91]	A, E	2014–2015	Thailand	Prevalence survey	A: gen	PCR	Rodents 0/270, water 0/100
Marinova-Petkova et al (2019) [92]	H, A, E	2017–2019	US Virgin Islands	Mixed	H: feb; A: gen	MAT/RDT	H 2/78
						MAT	Dogs 1/1
						PCR	H 1/2, dogs 0/1, water 1/5
Bakoss et al (2019) [93]	H, A	…	Slovakia	Cluster investigation	H: mix; A: gen	MAT	H 12/19, cows 9/15, rodents 2/44
Meny et al (2019) [94]	H, A, E	2015–2017	Uruguay	Prevalence survey	H: risk; A: gen	MAT/IIF	H 140/308
						MAT	Dogs 8/50, horses 11/22
						Culture	Water 6/25
Neela et al (2019) [95]	H, A, E	2016	Malaysia	Cluster investigation	H: feb; A: gen	MAT/RDT/ IgMELISA	H 4/12
						PCR	Rodents 6/12, water 6/18, soil 8/18
Nadia et al (2019) [96]	H, A	…	Malaysia	Prevalence survey	H: risk; A: gen	MAT	H 10/23, monkeys 5/10, shrews 1/1, rodents 4/43
Roqueplo et al (2019) [97]	H, A	2012–2014	Senegal	Prevalence survey	H: gen; A: gen	MAT	H 42/545, dogs 32/33, cows 17/56, goats 18/52, sheep 3/43, horses 16/20
						PCR	Rodents 2/36
Verma et al (2019) [98]	A, E	2016–2017	USA	Prevalence survey	A: gen	MAT	Cows 7/21, horses 13/31
						PCR	Rabbit 0/1, squirrels 0/1, shrews 3/6, rodents 60/93, water 2/89
Calderón et al (2019) [99]	H, A		Colombia	Prevalence survey	H: risk; A: risk	MAT	H 4/123, horses 130/153
						Culture	Horses 99/153
						PCR	Horses 0/99+
Mgode et al (2019) [100]	H, A		Tanzania	Prevalence survey	H: mix A: gen	MAT	H 72/455, shrews 1/5 rodents 3/21
						Culture	Shrews 0/5, rodents 0/21
Goh et al (2019) [21]	H, A	…	Malaysia	Prevalence survey	H: risk; A: gen	MAT	H 67/194, dogs 70/266, cats 7/47
Rodriguez et al (2020) [101]	H, A		Colombia	Prevalence survey	H: gen; A: gen	IgMELISA	H 25/83
						PCR	Rodents 4/53
Murcia et al (2020) [102]	H, A		Colombia	Prevalence survey	H: risk; A: risk	MAT	H 2/69, dogs 53/92
						Culture	Dogs 54/92
Alashraf et al (2020) [103]	H, A	…	Malaysia	Prevalence survey	H: risk; A: gen	MAT	H 5/58, dogs 26/127, cats 7/47
Grimm et al (2020) [104]	A, E	2008–2009	USA	Longitudinal monitoring	A: gen	MAT	Feral cats 0/19, opossums 60/112, racoons 121/221
						PCR	Water 6/8
Wójcik-Fatla et al (2020) [105]	A, E		Poland	Prevalence survey	A: gen	ELISA	Cows 0/80, pigs 51/86
						PCR	Air 2/50
Dushyant et al (2020) [106]	A, E		India	Prevalence survey	A: mix	Culture	Dogs 0/5, cows 0/77, rodents 0/5, water 0/3
						PCR	Dogs 0/10, cows 55/299, water 0/16, soil 0/4
Van et a (2017) [107]	H, A, E		Thailand	Prevalence survey	H: gen; A: gen	ImmunoC	H 199/280
						PCR	Fish 8/11, water 4/12, soil 9/12
Ospina-Pinto and Hernández-Rodríguez (2021) [108]	A, E	2019	Colombia	Prevalence survey	A: gen	MAT	Pigs 58/65
						Culture	Pigs 10/65, water 10/15
						PCR	Pigs 10/10+, water 10/10+
Benitez et al (2021) [109]	H, A	2015–2016	Brazil	Prevalence survey	H: gen; A: gen	MAT	H 11/597, dogs 155/729
Mgode et al (2021) [110]	H, A	…	Tanzania	Prevalence survey	H: feb; A: gen	MAT	H 15/50, goats 28/45, sheep 34/56, rodents 7/45
Machado et al (2021) [111]	H, A	2016–2018	Brazil	Prevalence survey	H: risk; A: gen	MAT	H 0/49, dogs 18/170, boars 9/74
Dreyfus et al (2021) [112]	H, A	2015	Bhutan	Prevalence survey	H: gen; A: gen	MAT	H 14/864, dogs 40/84, cows 48/130
Msemwa et al (2021) [113]	H, A	2018	Tanzania	Prevalence survey	H: risk; A: gen	MAT	H 33/205, dogs 66/414
Medkour et al (2021) [114]	H, A	2016–2019	Congo Algeria Senegal Djibouti	Prevalence survey	H: gen; A: gen	PCR	H 31/38, Gorilla 46/172
Shamsusah et al (2021) [115]	A, E	2017–2018	Malaysia	Prevalence survey	A: gen	PCR	Gorilla 1/12, rodents 1/23, soil 22/123, water 7/37
Aghamohammad et al (2022) [116]	H, A	2019	Iran	Prevalence survey	H: risk; A: mix	IgGELISA	H 1/51, cows 0/30, goats 1/31, sheep 2/30
Setyaningsih et al (2022) [117]	H, E	2017–2018	Indonesia	Case control	H: mix	PCR	H 34 (in case-control study^b^), water 6/100
de Souza Rocha et al (2022) [118]	H, A	2014–2015	Brazil	Prevalence survey	H: gen; A: gen	MAT	H 15/80, dogs 31/85, cats 0/10
						PCR	Dogs 13/68
Cunha et al (2022) [119]	H, A	2017–2019	Brazil	Prevalence survey	H: risk; A: risk	MAT	H 0/19, dogs 16/264
Richard et al (2022) [120]	A, E	2018–2020	France	Longitudinal monitoring	A: gen	PCR	Rodents 68/189, water 158/1031
do Couto (2022) [121]	H, A	…	Brazil	Prevalence survey	H: risk; A: risk	MAT	H 0/200, dogs 5/40
Meny et al (2022) [122]	H, A	2017–2020	Uruguay	Prevalence survey	H: risk; A: gen	MAT	H 6/150, horses 546/891

Abbreviations: A, animals; ALR, agglutination-lyse reaction; CAR, cross-agglutination reaction; E, environment; ELISA, enzyme-linked immunosorbent assay; exp, exposed; feb, febrile; gen, general; H, humans; Ig, immunoglobulin; IIF, indirect immunofluorescence; ImmunoC, immunochromatography; MAT, microscopic agglutination test; mix, mixed; NA, not available; PCR, polymerase chain reaction; RDT, rapid diagnostic test; risk, at risk;

^a^Plus signs (+) indicate that tested samples were positive to a previous test.

^b^Case-control study without number of all tested patients.

### Sampling and Study Designs

*Homo sapiens* was the most studied species, with a median number of 160 samples (range, 10–24 990), followed by dogs (Supplementary Table 3). Domestic animals had a median of 219 samples (range, 1–2 299 209); wild animals, a median of 88 (5–2820); and the environment, a median of 47 (1–1031).

Of the studies, 70.6% were cross-sectional, and 23.5% were repeated cross-sectional. Only 6 studies (5.9%) were longitudinal, including 2 serological human-animal [46, 47] and 4 animal-environment studies. The predominant study design was prevalence survey (n = 70 [68.6%]) (Supplementary Figure 3A). Most cluster investigations (10 of 14) were initiated after only human cases. Samples from different One Health compartments originated from the same household in 17 studies (16.7%) and from the same establishment in 25 (24.5%) (eg, farms [39, 42, 43, 48, 60, 63, 69, 75, 79, 80, 93, 94, 106, 108], zoos [52, 54, 115], slaughterhouses [86, 116], police stations [99, 102], or shelters [21, 103]). More than half of the studies (n = 60 [58.8%]) did not define the spatial distance between compartments, and temporal distances were often inadequately described, with 24 studies (23.5%) failing to specify the study date.

Among the 70 prevalence surveys, 56 (80%) did not specify a sample size calculation, 9 provided partial calculation, and 5 conducted complete calculation for each species [56, 91, 100, 102, 108]. Only 9% of the prevalence studies (6 of 70) were fully randomized, with partial randomization in 10% (7 of 70) and 81% (57 of 70) not randomized at all.

The febrile population was the most common in human studies (n = 27 [32%]), while the general population was most common in animal studies (Supplementary Figure 3B). Twenty-six human studies (29%) targeted at-risk populations, including animal care workers [21, 38, 52, 54, 96, 103, 113, 122], farmers [37, 39, 48, 60, 63, 75, 79], meat industry workers [41, 86, 116], security personnel handling animals [99, 102], miners [70], people engaged in high-risk practices like hunting [111] or animal hoarding disorder [119], and homeless people owning animals [121]. In animal studies, only 4% targeted at-risk populations, including police-owned horses [99] and dogs [102].

### Diagnostic Methods

The MAT was the predominant diagnostic method, in 88% of human studies (74 of 84) and 76% of animal studies (74 of 98) (Supplementary Figure 3C). Sixty-six studies used this test for both humans and animals. Threshold titers for positive MAT results varied significantly across studies, ranging from 20 [100, 110] to 500 [49], and 14 studies had different thresholds depending on the species [21, 30, 34, 39, 40, 43, 46, 49, 59, 71, 76, 86, 87, 99]. Six studies used 4-fold titer increase to diagnosis [38, 49, 59, 81, 83, 95]. The number of antigens tested ranged from 2 representing 2 serogroups [79] to 39 covering 23 [86], compared to the World Health Organization's recommended 19 antigens across 16 serogroups as of 2003 [123]. Through 2003, the median was 10 antigens (interquartile range, 7–12) and 9 serogroups (6–12); after 2003, it increased to 15 antigens (10–23) and 13 serogroups (9–19). The inclusion of serovar Patoc, recommended for its cross-reactivity with pathogenic serogroups [123, 124], was reported in 27% (6 of 22; 3 missing values) of studies before 2003 and 34% (20 of 59; 3NA) thereafter.

A quarter of studies integrated various tests (additional biospecimen details in Supplementary Table 4). A minority of studies in humans (13%) and animals (33%) used molecular approaches that can provide more precise insight into the infecting strains.

### Risk Factors

Sociodemographic or animal data were collected in more than half of the studies (n = 60). Statistical analyses of data were conducted in 39 studies (38.2%). One third of studies used χ² and/or Fisher tests, and another third used multivariate logistic regression [21, 39, 81, 83, 90, 94, 99, 102, 104, 109, 112, 113, 122]. The majority of studies conducting statistical analysis (n = 29) investigated One Health compartments interactions, notably between humans and animals, and human and the environment (Supplementary Table 5).

Animal contact was the most frequent risk factor identified for humans (Supplementary Table 6). Eight studies highlighted occupational risks including livestock farming [39, 94] and field working [43, 83, 117]. At-risk practices were identified, such as time spent in rodent-infested houses [112], poor food and waste management [99, 117], and a high number of outings for animals [99].

### Limitations and Recommendations

Limitations related to MATs, such as lack of sensitivity or suitability to identify serovars, were cited in 24.5% of studies. Issues regarding false-negatives were cited in 22.5% of studies, and small sample sizes were reported in 12.7%. More than half of studies did not report any limitations. Recommendations by authors included increased leptospirosis awareness (26.5%), better hygiene practices (21.6%), more local bacterial strain identification (15.7%), routine diagnostic inclusion (13.7%), and enhanced animal vaccination (8.8%).

### Study Quality

Study quality assessed via the JBI tool showed mean scores of 0.47 (95% CI, .44–.50) for humans, 0.47 (.43–.50) for domestic animals, 0.64 (.60–.67) for wild animals, and 0.79 (.76–.83) for the environment. Study design mean scores ranged from 0.50 to 0.66. In prevalence surveys, 51% of JBI scores exceeded 0.5. Full details are provided in Supplementary Figure 4.

### Meta-Analyses and Meta-Regressions

**Figure 3.**
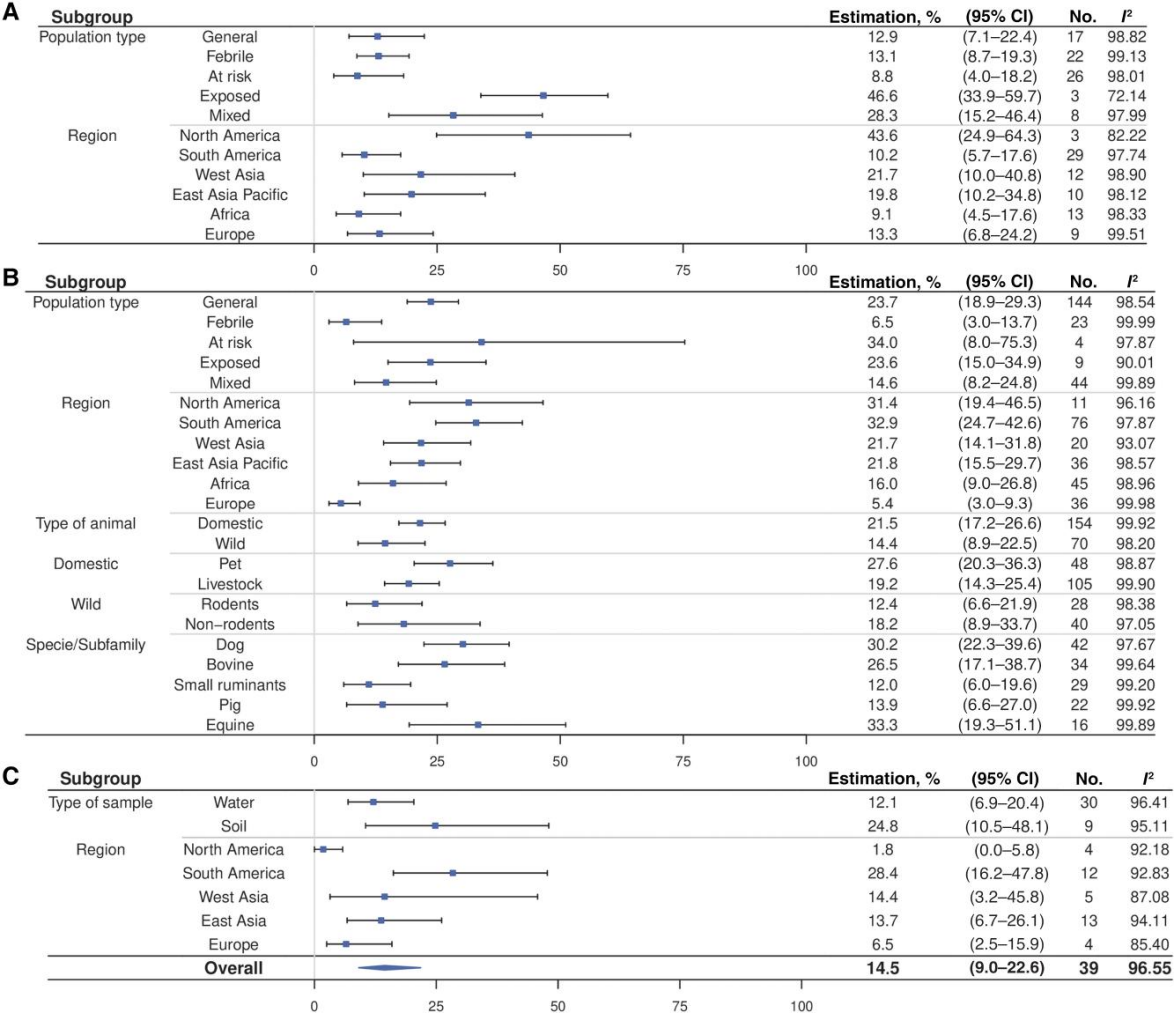
Meta-analysis of human serology, animal serology and environmental positivity rate. *A*, Forest plot for human serology. *B*, Forest plot for animal serology. *C*, Forest plot for environmental positivity rate. The overall prevalence in humans and animals was not calculated because of the variety of population types included. A sample from multiple domestic species without distinction was not included for either pet or livestock. Two samples from multiple wild species without distinction were not included in either rodent or nonrodent categories. Results from the air sampling were not include in the meta-analysis because of difficulties of comparability with water and soil. Africa does not appear for the environment because there only 1 occurrence. Abbreviation: CI, confidence interval.

The meta-analysis revealed that the exposed population had a higher seroprevalence than the general population (Figure 3*A* and Supplementary Table 7*A*). No difference in seroprevalence was identified among the occupations and lifestyles of the at-risk population (Supplementary Table 7*B*). About the geographic situation, East Asia Pacific (*P* = .02), West Asia (*P* = .046) and North America (*P* = .02) showed significantly higher seroprevalence (Supplementary Table 7*C*). In addition, Thailand (*P* = .005), Malaysia (*P* = .12), India (*P* = .01), the United States (*P* = .02), and Colombia (*P* = .01) had higher seroprevalence (Supplementary Table 7*D*).

For animal seroprevalence, the general population exhibited a higher rate, (23.7% [95% CI, 18.9–29.3]) than the febrile population (6.5% [3.0–13.]) (*P* < .001) (Figure 3*B* and Supplementary Table 8*A*). Europe had the lowest seroprevalence, significantly lower than the 5 other regions (Supplementary Table 8*B*). Argentina (*P* = .048) and Belize (*P* = .01) had higher seroprevalence (Supplementary Table 8*C*). No significant differences were found within the various animal categories (Supplementary Tables 8*D*–8*AF*). Dogs (*P* = .005), cows (*P* = .01), horses (*P* = .005), poultry (*P* = .04), foxes (*P* = .37), and monkeys (*P* = .03) had higher seroprevalence than rodents (Supplementary Table 8*G*).

Environmental sample positivity was 14.5% (95% CI, 9.0–22.6) (Figure 3*C*), with no significant difference between water and soil (*P* = .17) (Supplementary Table 9*A*). North America (*P* = .08) and Europe (*P* = .03) were identified as having a lower seroprevalence than South America (Supplementary Table 9*B*).

**Figure 4.**
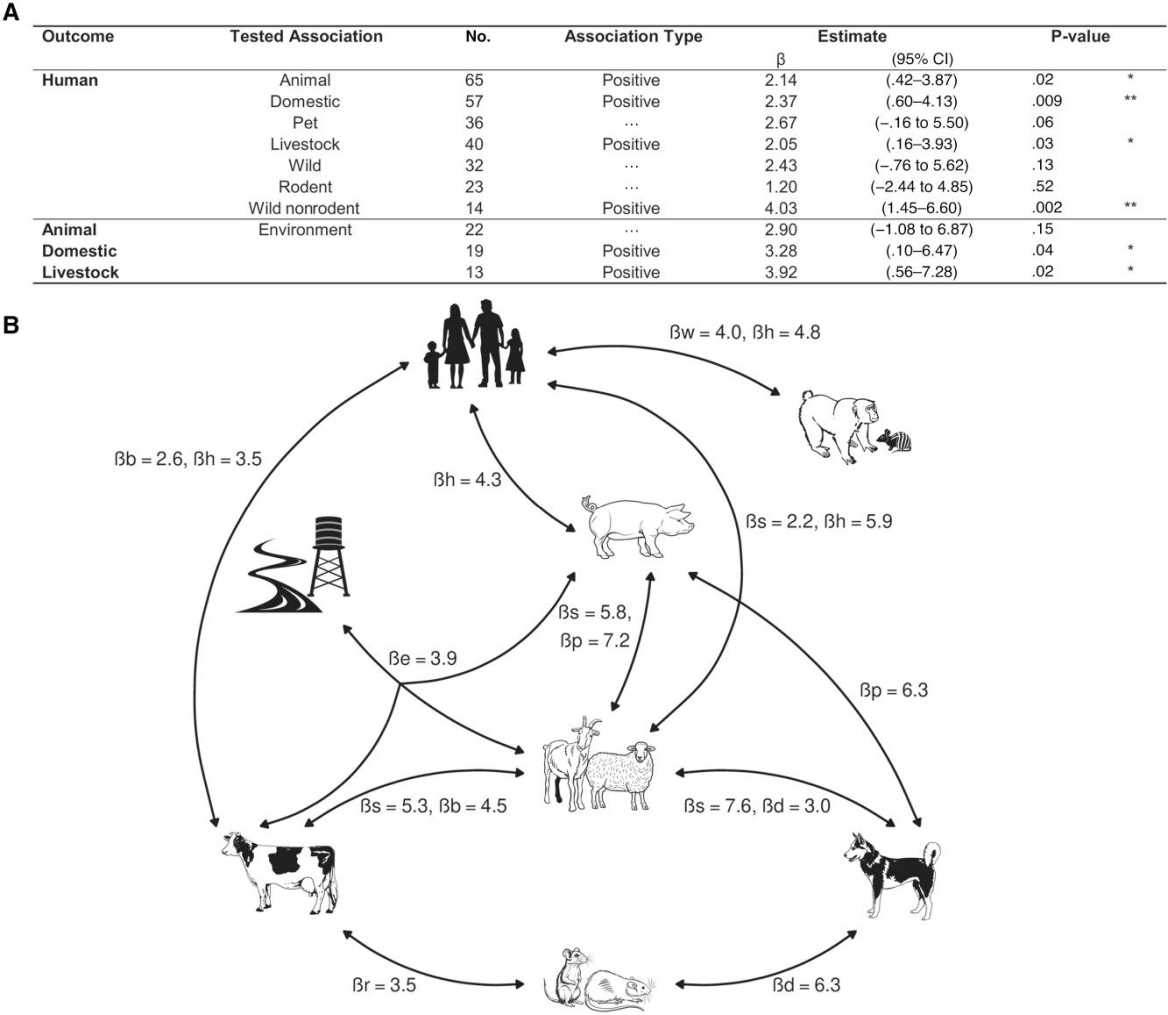
Interconnections between compartments. *A*, Meta-regression analyses of human seroprevalence explained by animal seroprevalence and animal seroprevalence explained by environmental positivity rate, with seroprevalence defined as every outcome and association tested for in humans and animals. *P < .05; **P < .01. Abbreviation: CI, confidence interval. *B*, Significant links of *Leptospira* presence or exposure identified by the overview of One Health studies, with presentation of β coefficients of variable with significant effects generated by meta-regressions. Abbreviations: b, bovine seroprevalence; d, dog seroprevalence; h, human seroprevalence; l, livestock seroprevalence; p, pig seroprevalence; r, rodent seroprevalence; s, small ruminant seroprevalence; w, wild nonrodent seroprevalence.

Meta-regression showed a positive association between human and animal seroprevalences (*P* = .02) (Figure 4*A*). Human seroprevalence was positively associated with that of domestic animal (*P* = .009), livestock (*P* = .03), and wild nonrodents (*P* = .002). Some livestock species including cows (*P* = .008), pigs (0.02), and small ruminants (goats and sheep) (*P* = .007) were identified as being associated with humans (Supplementary Table 10*A*). Positive associations were found between environmental positivity rate and domestic animal seroprevalence (*P* = .04), particularly for livestock (*P* = .02), but no link was identified between humans and the environment (Supplementary Table 10*B*).

Seroprevalence links between various animals have been identified: bovines with rodents, rodents with dogs, and dogs with livestock, as well as within livestock species (Figure 4*B*). Results are detailed in Supplementary Table 10*C*. Explorations of PCR data, revealing a link between an animal's PCR result and its own seroprevalence, are presented in Supplementary Table 11.

## DISCUSSION

Our systematic review highlights a significant gap in comprehensive One Health studies on leptospirosis, particularly noting the scarcity of studies incorporating the environmental compartment. This deficiency restricts understanding of the full transmission dynamics of this zoonotic disease. While 94.2% of studies included animals and 82.4% included humans, only 31.4% incorporated environmental factors. Furthermore, only 9.8% of the studies explored all 3 compartments, a trend also observed in One Health networks [125] and reviews of other multicompartmental public health issues like antimicrobial resistance [126–130], salmonellosis [131], and giardiasis [132]. Reviews of leptospirosis in Africa also show a predominance of animal studies, with multicompartmental studies representing only 6.5% of studies [133–136]. One Health's historical focus on human-animal interactions has gradually expanded to include environmental aspects [137], but dynamic environmental complexities [11] and anthropocentrism [125] may have limited this inclusion. In recent years, more attention has been given to the environment, aided by developments like selective media for growing *Leptospira*, which allow easier bacteria isolation [138] and increased awareness of climate change's impact on environmental factors, anchoring the environmental compartment into the One Health approach in the future [11].

Methodological issues were widespread within the studies examined. Only 7 studies used longitudinal monitoring, limiting the potential to understand the disease's dynamics across transmission pathways and seasonal variations, mirroring gaps in One Health research on antimicrobial resistance [126]. In addition, most prevalence surveys lacked adequate sample size calculations, and many did not use randomized sampling strategies. Unclear spatiotemporal distances between sampling units in different compartments may complicate conclusions about transmission routes. Moreover, many studies lacked detailed sociodemographic or animal collected data and comprehensive statistical analyses. The methodological shortcomings, reflected in low JBI scores, underscore an urgent need for improved, more rigorous, study designs and analytical methods in One Health research. A standardized, peer-reviewed tool tailored to evaluate One Health studies is also necessary. Indeed, the JBI evaluation tool used here is designed for prevalence studies and was not well suited to all the studies included in this review, especially those focused on environmental or wildlife research. This highlights the need for more tailored assessment tools specifically designed for environmental and wildlife protocols. This resulted in the exclusion of risk of bias scoring as a criterion for inclusion in the meta-analysis.

Addressing critical questions regarding the connections between human, animal, and environmental contamination requires comprehensive longitudinal molecular studies or rigorously designed multicompartmental cross-sectional research to investigate spatial correlations between compartments. These studies would not only help determine whether the same *Leptospira* strains are transmitted between compartments but would also pinpoint the primary sources of contamination.

Our meta-analysis revealed a significant positive association between human and animal seroprevalences and with animal seroprevalence and environmental positivity rate. This highlights the interconnectedness of humans, animals, and the environment in leptospirosis spread and underscores the need for more rigorous One Health research methods.

Human seroprevalence was associated with seroprevalence in livestock (cows, pigs, and small ruminants) but not with canine seroprevalence, suggesting that livestock's denser populations and larger daily urine excretion, along with larger leptospiruria [139], enhance pathogen spread. This supports the hypothesis that the risk of transmission to a human from a dog is low [140]. However, links between seroprevalence in dogs and that in rodents and small ruminants have been demonstrated. In addition, there were links between livestock and the environment, and between humans and wildlife. These connections can reveal a transmission network and simultaneous contaminations.

Our meta-analysis also revealed a lower seroprevalence in febrile animals, potentially due to early sampling before antibody formation. Higher seroprevalences were mostly identified in South America and Asia across the 3 compartments, coinciding with estimates of leptospirosis morbidity [4]. However, it is crucial to note that these regional or species-type estimates are derived from studies with varying designs and populations. Therefore, they should not be considered as replacements for targeted, ad hoc prevalence estimates.

Using seroprevalence as a variable in meta-regressions can bias interpretations with false-negatives, particularly for healthy carrier animals like rodents. Implementing a longitudinal design with 3-month intervals between sample collections could minimize underestimation in serology [141, 142]. However, there were only 2 longitudinal studies of 89 studies with serology [46, 61]. Moreover, the MAT, widely used, is the main source of data for the meta-analysis, while it has limitations in estimating seroprevalence, especially regarding sensitivity and subjective interpretation. World Health Organization recommendations, in 2003 to include 19 serovars representing 16 serogroups [123] and the Patoc serovar, which cross-reacts with several antigens, have been partially followed, affecting the test's robustness. Indeed, the median number of antigens was 15 (interquartile range, 10–23) after 2003, and only 34% of MAT studies used the Patoc serovar.

These challenges in implementing leptospirosis diagnosis make it impossible for some countries to detect cases. Using MAT with a limited number of serovars or missing key serovars can result in underestimation. Both biases distort our understanding of global leptospirosis distribution. Despite these challenges, MAT remains a primary diagnostic tool, though accuracy could be improved by integrating additional tests and Bayesian classification with clinical data [7]. Knowledge of local serovars is crucial for effective surveillance, improving MAT's sensitivity, and allowing optimization of vaccination strategies, as demonstrated by successful vaccination programs in New Zealand during the 1970s and 1980s [143], but a minority of studies used a molecular approach.

Our meta-analysis faced limitations due to the small number and size of included studies, preventing certain meta-regressions and diminishing the robustness of our findings. The limited number of studies included made some analyses unfeasible, such as advanced analyses of the environmental compartment. A precision failure on spatiotemporal distance between compartments reduced the robustness of our hypotheses about transmissions. Moreover, it is clear that the lack of rigor, particularly the absence of randomization, in the data collection used for the meta-analysis leads to problems of representativeness and therefore weakens the robustness of the results. These methodological weaknesses are reflected in low JBI scores, indicating a high risk of bias, and high *I*² values suggest considerable heterogeneity among studies, complicating interpretation, but a high *I*² value does not necessarily reflect overly heterogeneous and nonpoolable data [144, 145].

Despite these issues, we adjusted for population types in our meta-regression models to maintain biological relevance and methodological consistency. Associations identified should be considered with caution; they are probable hypotheses that need to be confirmed with rigorous protocols. However, to compensate for the lack of representativeness of the studies used to measure associations between compartments, we explored the association between human seroprevalence and that in animals, more specifically domestic animals, in a subset presenting a more rigorous methodology than most of the included studies. A small sample of randomized human and animal studies also showed a positive association between human and animal seroprevalences, but this was not significant (Supplementary Table 12). The fact that similar results are obtained when including only more robust studies helps support our results. In addition, due to our chosen eligibility criteria, our review did not consider multicompartmental studies that presented results from various compartments in different publications, potentially limiting the evidence already available.

The choices and strategy in our review aimed to evaluate the ability of multicompartmental field-collected data to assess transmission risks and patterns. Other approaches, such as combining unicompartmental data and existing phylogenetic data available in specific geographic areas, not included in our review, have shown their value [146, 147]. While our focus was on applying One Health approach using prevalence data across samples from multiple compartments, other methods, such as using questionnaires to gather animal contact data or models incorporating environmental data, are steps toward a One Health approach.

In conclusion, our systematic review and meta-analysis highlighted significant gaps in One Health research, particularly the need for more comprehensive studies that include environmental factors alongside human and animal factors. The challenges of laboratory diagnostics and the need for larger, more rigorous studies are evident. We advocate for the One Health approach to better understand and manage leptospirosis, emphasizing the need for holistic studies to accurately determine disease prevalence, ecology, and transmission routes. This approach should be well supported, and high methodological standards should be maintained to ensure reliable outcomes and effective interventions. Ultimately, this strategy will allow for the development of targeted prevention, enhanced surveillance systems, improved diagnostics, and effective vaccination campaigns, all grounded in a thorough understanding of the epidemiological interactions between humans, animals and their environments.

## Supplementary Material

ofae757_Supplementary_Data

## Supplementary Data

Supplementary materials are available at *Open Forum Infectious Diseases* online. Consisting of data provided by the authors to benefit the reader, the posted materials are not copyedited and are the sole responsibility of the authors, so questions or comments should be addressed to the corresponding author.
