# Supplementary material for: One Health Field Approach Applied to Leptospirosis: A Systematic Review and Meta-Analysis Across Humans, Animals and the Environment
Source: Open Forum Infect Dis. 2024 Dec 30;12(1):ofae757. doi: 10.1093/ofid/ofae757 (PMC11752865; doi:10.1093/ofid/ofae757)
Supplement: ofae757_Supplementary_Data [file ofae757_supplementary_data.docx]

Contents

[Supplementary Material 1: Search strategy. 3](#_Toc183016909)

[Search through Web of Science. 3](#_Toc183016910)

[Table S1: Reason for exclusion for 10 example items 3](#_Toc183016911)

[Supplementary Material 2: JBI assessment. 5](#_Toc183016912)

[Figure S1: JBI critical appraisal checklist for studies reporting prevalence data. 5](#_Toc183016913)

[Figure S2: JBI critical appraisal checklist for case control studies. 6](#_Toc183016914)

[Supplementary Material 3: Geographical repartition of studies and languages. 7](#_Toc183016915)

[Table S2: Number of studies by region and number of studies by country. 7](#_Toc183016916)

[Supplementary Material 4: Sample sizes. 9](#_Toc183016917)

[Table S3 Median number of individuals sampled per study with the number of studies involved. 9](#_Toc183016918)

[Supplementary Material 5: Description of studies included. 10](#_Toc183016919)

[Figure S3: Description of studies included. (A) Design distribution. (B) Proportions of types of study populations for humans and animal studies. (C) Number of studies using each test or combination of tests in (1) human, (2) animal and (3) environmental sampling. 10](#_Toc183016920)

[One study in human (MAT+Culture+Other) does not appear. 10](#_Toc183016921)

[Supplementary Material 6: Biospecimen. 11](#_Toc183016922)

[Table S4: Biospecimen used for human and animal tests. 11](#_Toc183016923)

[Supplementary Material 7: Searching for links between One Health compartments. 12](#_Toc183016924)

[Table S5: Statistical methods looking for links between One Health components. 12](#_Toc183016925)

[Table S6: Risk factors identified using a statistical approach by the studies. 14](#_Toc183016926)

[Supplementary Material 8: Risk of bias assessment results. 16](#_Toc183016927)

[Figure S4: Mean score by study design and compartment. 16](#_Toc183016928)

[Supplementary Material 9: Meta-regressions with human seroprevalence outcome. 17](#_Toc183016929)

[Table S7a: Meta-regression with mixed-effects model, effect of the type of study population on human seroprevalence. 17](#_Toc183016930)

[Table S7b: Meta-regression with mixed-effects model, effect of type of risk the on human at risk seroprevalence. 17](#_Toc183016931)

[Table S7c: Meta-regression with mixed-effects model, effect of the region on human seroprevalence, adjusted on study population type. 17](#_Toc183016932)

[Table S7d: Meta-regression with mixed-effects model, effect of the country on human seroprevalence, adjusted on study population type. 18](#_Toc183016933)

[Supplementary Material 10: Meta-regressions with animal seroprevalence outcome. 19](#_Toc183016934)

[Table S8a: Meta-regression with mixed-effects model, effect of the type of study population on animal seroprevalence. 19](#_Toc183016935)

[Table S8b: Meta-regression with mixed-effects model, effect of the region on animal seroprevalence, adjusted on study population type. 19](#_Toc183016936)

[Table S8c: Meta-regression with mixed-effects model, effect of the country on animal seroprevalence, adjusted on study population type. 20](#_Toc183016937)

[Table S8d: Meta-regression with mixed-effects model, effect of the animal type on animal seroprevalence, adjusted on study population type. 21](#_Toc183016938)

[Table S8e: Meta-regression with mixed-effects model, effect of the animal type on domestic animal seroprevalence, adjusted on study population type. 21](#_Toc183016939)

[Table S8f: Meta-regression with mixed-effects model, effect of the animal type on wild animal seroprevalence, adjusted on study population type. 21](#_Toc183016940)

[Table S8g: Meta-regression with mixed-effects model, effect of the specie on animal seroprevalence, adjusted on study population type. 22](#_Toc183016941)

[Supplementary Material 11: Meta-regressions with environmental positive rate outcome. 23](#_Toc183016942)

[Table S9a: Meta-regression with mixed-effects model, effect of the nature of sampling on environmental positive rate. 23](#_Toc183016943)

[Table S9b: Meta-regression with mixed-effects model, effect of the region on environmental positive rate. 23](#_Toc183016944)

[Supplementary Material 12: Meta-regression models seeking for links between compartments. 24](#_Toc183016945)

[Table S10a: Meta-regression analyses seeking for associations between human and animal seroprevalences. 24](#_Toc183016946)

[Table S10b: Meta-regression analyses seeking for associations between the environment positivity rate and animal and human seroprevalences. 25](#_Toc183016947)

[Table S10c: Meta-regression analyses seeking for associations between seroprevalences of different animal categories. 25](#_Toc183016948)

[Supplementary Material 13: Exploration of PCR data. 27](#_Toc183016949)

[Table S11: Meta-regression analyses of PCR results explained by various positivity rate. 27](#_Toc183016950)

[Supplementary Material 14: Exploration to validate the results of the meta-analysis. 27](#_Toc183016951)

[Table S12: Meta-regression analyses among subsets with a potentially better representativeness. 27](#_Toc183016952)

# Supplementary Material 1: Search strategy.

## Search through Web of Science.

[(leptospirosis OR leptospira) AND “One Health” AND (sample OR sera)) OR ((leptospirosis OR leptospira) AND (human OR people OR resident OR individual ) AND ( animal OR mammal OR livestock ) AND (sample OR sera)) OR ((leptospirosis OR leptospira) AND (human OR people OR resident OR individual) AND (environment OR soil OR water) AND (sample OR sera)) OR ((leptospirosis OR leptospira) AND (environment OR soil OR water) AND (animal OR mammal OR livestock) AND (sample OR sera)].

## Table S1: Reason for exclusion for 10 example items

| Study | Reason for exclusion | Classification |
| --- | --- | --- |
| Genetic Evidence for a Potential Environmental Pathway to Spillover Infection of Rat-Borne Leptospirosis.  *Casanovas-Massana A, de Oliveira D, Schneider AG, Begon M, Childs JE, Costa F, Reis MG, Ko AI, Wunder EA. J Infect Dis. 2022* | No original data from sampling carried out for the study  Number of tested not shown | Wrong study type |
| Despite high-risk exposures, no evidence of zoonotic transmission during a canine outbreak of leptospirosis.  *Guagliardo SAJ, Iverson SA, Reynolds L, Yaglom H, Venkat H, Galloway R, Levy C, Reindel A, Sylvester T, Kretschmer M, LaFerla Jenni M, Woodward P, Beatty N, Artus A, Klein R, Sunenshine R, Schafer IJ. Zoonoses Public Health. 2019* | Presentation of samples from a single compartment: human  Number of tested shown only for humans | Unicompartmental survey: human |
| Are Small Animal Practitioners Occupationally Exposed to Leptospirosis? Results of a Serological Survey.  *Mazzotta E, Lucchese L, Salata C, Furlanello T, Baroni E, Zotti A, Venturi G, Fincato A, Marchione S, Capello K, Natale A. Int J Environ Res Public Health. 2022* | Presentation of samples from a single compartment: human  Number of tested shown only for humans | Unicompartmental survey: human |
| Leptospira diversity in animals and humans in Tahiti, French Polynesia.  *Guernier V, Richard V, Nhan T, Rouault E, Tessier A, Musso D. PLoS Negl Trop Dis. 2017* | Presentation of samples from a single compartment: animal | Unicompartmental survey: animal |
| Human Leptospirosis on Reunion Island, Indian Ocean: Are Rodents the (Only) Ones to Blame?  Guernier V, Lagadec E, Cordonin C, Le Minter G, Gomard Y, Pagès F, et al. PLoS Negl Trop Dis. 2016 | Presentation of samples from a single compartment: animal  Number of tested humans not shown | Unicompartmental survey: animal |
| Seeking the environmental source of Leptospirosis reveals durable bacterial viability in river soils.  Thibeaux R, Geroult S, Benezech C,  Chabaud S, Soupe-Gilbert M-E, Girault D, et al. PLoS Negl Trop Dis. 2017 | Presentation of samples from a single compartment: animal  Number of tested humans not shown | Unicompartmental survey: animal |
| Environmental DNA metabarcoding to detect pathogenic Leptospira and associated organisms in leptospirosis-endemic areas of Japan  Sato Y, Mizuyama M, Sato M, Minamoto T, Kimura R, Toma C. Sci Rep. 2019 | Presentation of samples from a single compartment: human  Number of tested shown only for the environment | Unicompartmental survey: environment |
| Molecular Detection and Typing of Pathogenic Leptospira in Febrile Patients and Phylogenetic Comparison with Leptospira Detected among Animals in Tanzania.  *Allan KJ, Maze MJ, Galloway RL, et al. Am J Trop Med Hyg. 2020* | Presentation of samples from a single compartment: human  Number of tested shown only for humans | Unicompartmental survey: human |
| Serological Survey of Veterinarians to Assess the Zoonotic Potential of Three Emerging Swine Diseases in Mexico. *Rivera-Benitez JF, Rosas-Estrada K, Pulido-Camarillo E, de la Peña-Moctezuma A, Castillo- Juarez H, Ramirez-Mendoza H. Zoonoses Public Health. 2014* | Presentation of samples from a single compartment: human  Number of tested shown only for humans | Unicompartmental survey: human |
| The occurrence and significance to animal health of Leptospira, Mycobacterium, Escherichia coli, Brucella abortus and Bacillus anthracis in sewage and sewage sludges.  [*Jones*](https://pubmed.ncbi.nlm.nih.gov/?term=Jones+PW&cauthor_id=6780619) *PW,*[*Rennison*](https://pubmed.ncbi.nlm.nih.gov/?term=Rennison+LM&cauthor_id=6780619) *LM,* [*Matthews*](https://pubmed.ncbi.nlm.nih.gov/?term=Matthews+PR&cauthor_id=6780619) *PRJ,*[*Collins*](https://pubmed.ncbi.nlm.nih.gov/?term=Collins+P&cauthor_id=6780619) *P,*[*Brown*](https://pubmed.ncbi.nlm.nih.gov/?term=Brown+A&cauthor_id=6780619) *A. J. Hyg. 1981* | Presentation of samples from a single compartment: human  Number of tested shown only for the environment | Unicompartmental survey: environment |

# Supplementary Material 2: JBI assessment.

All 9 questions were asked for studies on humans and on domestic animals, 8 questions (from 1 to 8) for those on wild animals and 5 questions (1-4-6-7-8) for those on the environment. For the only article evaluated by the case-control tool, all 10 questions were asked for the human compartment and 5 for the environment. Studies which included several species within a given group, were attributed only one score for that set of species. For those studies with several species of a given group, if the protocol differed between species, then the answer "yes" for at least one species to a question lead to the assignment of a “yes” answer to the question overall, whatever the responses for the other species. Inversely, a “no” answer was attributed to a question only if the answer was “no” for all the species in the study for the group considered.

## Figure S1: JBI critical appraisal checklist for studies reporting prevalence data.


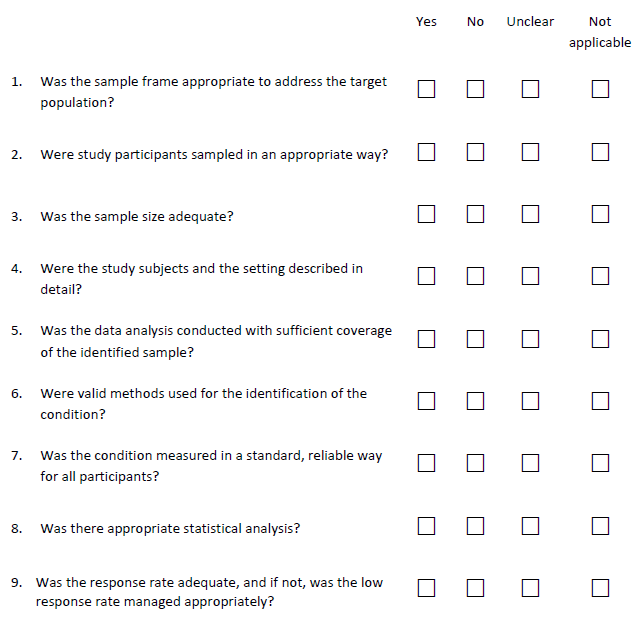


## Figure S2: JBI critical appraisal checklist for case control studies.


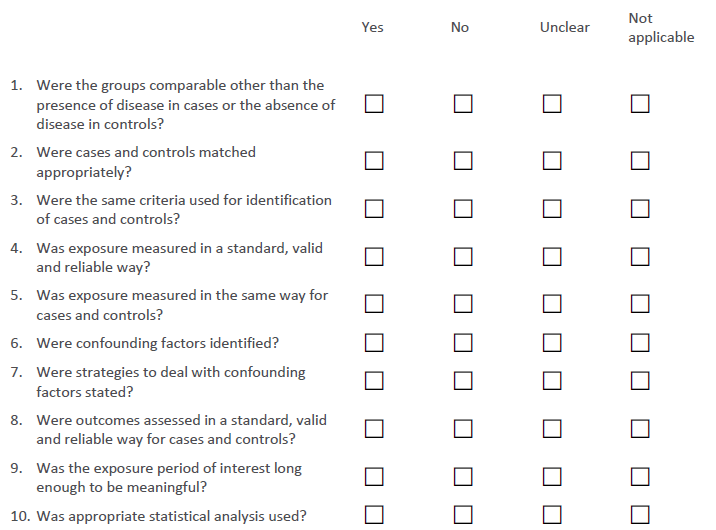


# Supplementary Material 3: Geographical repartition of studies and languages.

## Table S2: Number of studies by region and number of studies by country.

| Region (N studies) | Country | N studies |
| --- | --- | --- |
| North America (6) | Mexico | 2 |
|  | U.S.A. | 4 |
| South America (36) | Argentina | 1 |
|  | Belize | 1 |
|  | Bolivia | 1 |
|  | Brazil | 12 |
|  | Colombia | 12 |
|  | Ecuador | 1 |
|  | Nicaragua | 1 |
|  | Peru | 2 |
|  | Saint Kitts and Nevis | 1 |
|  | Uruguay | 3 |
|  | U.S. Virgin Islands | 1 |
| West Asia (15) | Bhutan | 1 |
|  | India | 10 |
|  | Iran | 3 |
|  | Nepal | 1 |
| East Asia Pacific (19) | China | 2 |
|  | Indonesia | 1 |
|  | Malaysia | 7 |
|  | New-Zealand | 1 |
|  | Philippines | 1 |
|  | Taiwan | 1 |
|  | Thailand | 6 |
| Africa (13) | Algeria, Congo, Djibouti, Senegal | 1 |
|  | Cape Verde | 1 |
|  | Egypt | 3 |
|  | Madagascar | 1 |
|  | Senegal | 1 |
|  | Seychelles | 1 |
|  | Tanzania | 5 |
| Europe (13) | Croatia | 2 |
|  | France | 2 |
|  | Germany, Luxembourg | 1 |
|  | Italia | 2 |
|  | Poland | 1 |
|  | Slovakia | 3 |
|  | Ukraine | 2 |

Twenty-three studies were published in languages other than English, including Spanish (11), Portuguese (3), Chinese (2), Indonesian (1), German (1), Russian (1), Croatian (1), Italian (1), Slovakian (1), and French (1).

# Supplementary Material 4: Sample sizes.

## Table S3 Median number of individuals sampled per study with the number of studies involved.

|  |  | **Median (Min - Max)** | **N studies** |
| --- | --- | --- | --- |
| Humans |  | 160 (10 - 24,990) | 84 |
| Domestic animals | Dog | 61 (1 - 4,369) | 50 |
|  | Bovine | 155 (1 - 22,669) | 42 |
|  | Pig | 129 (13 - 989,659) | 26 |
|  | Equine | 60 (1 - 70,674) | 20 |
|  | Goat | 52 (1 – 1,639) | 16 |
|  | Sheep | 80 (30 – 16,278) | 14 |
|  | Goat+Sheep | 20,957 (162 – 41,752) | 2 |
|  | Cat | 10 (1 - 1,238,876) | 9 |
|  | Camel | 36 (22 - 50) | 2 |
|  | Poultry | 37 (37 - 37) | 1 |
|  | **Total** | 219 (1 - 2,299,209) | 76 |
| Wild animals | Rodents | 64 (1 - 2,820) | 48 |
|  | Shrew | 8 (1 - 68) | 8 |
|  | Monkey | 11 (1 - 172) | 6 |
|  | Bandicoot | 11 (9 - 24) | 3 |
|  | Boar | 74 (1 - 459) | 3 |
|  | Deer | 567 (1 – 1,133) | 3 |
|  | Fox | 11 (2 - 70) | 3 |
|  | Rabbit | 1 (1 - 1) | 2 |
|  | Tatou | 10 (4 - 16) | 2 |
|  | Feral cat | 10 (1 – 19) | 2 |
|  | Opossum | 56 (1 - 112) | 2 |
|  | Non-specified | 74 (49 – 100) | 2 |
|  | Snake | 55 (1 - 110) | 2 |
|  | Fish | 18 (11 - 25) | 2 |
|  | Bat | 9 ( - ) | 1 |
|  | Bird | 143 ( - ) | 1 |
|  | Wolf | 4 ( - ) | 1 |
|  | Coati | 3 ( - ) | 1 |
|  | Lion | 2 ( - ) | 1 |
|  | Zebra | 2 ( - ) | 1 |
|  | Civet | 2 ( - ) | 1 |
|  | Squirrel | 6 (1 - 11) | 1 |
|  | Racoon | 221 ( - ) | 1 |
|  | Mole | 1 ( - ) | 1 |
|  | Marmot | 120 ( - ) | 1 |
|  | Wolf | 4 ( - ) | 1 |
|  | **Total** | 92 (5 - 2,820) | 56 |
| Environment | Water | 40 (1 - 1,031) | 31 |
|  | Soil | 60 (4 - 292) | 9 |
|  | Air | 50 ( - ) | 1 |
|  | **Total** | 47 (1 - 1,031) | 32 |

# Supplementary Material 5: Description of studies included.


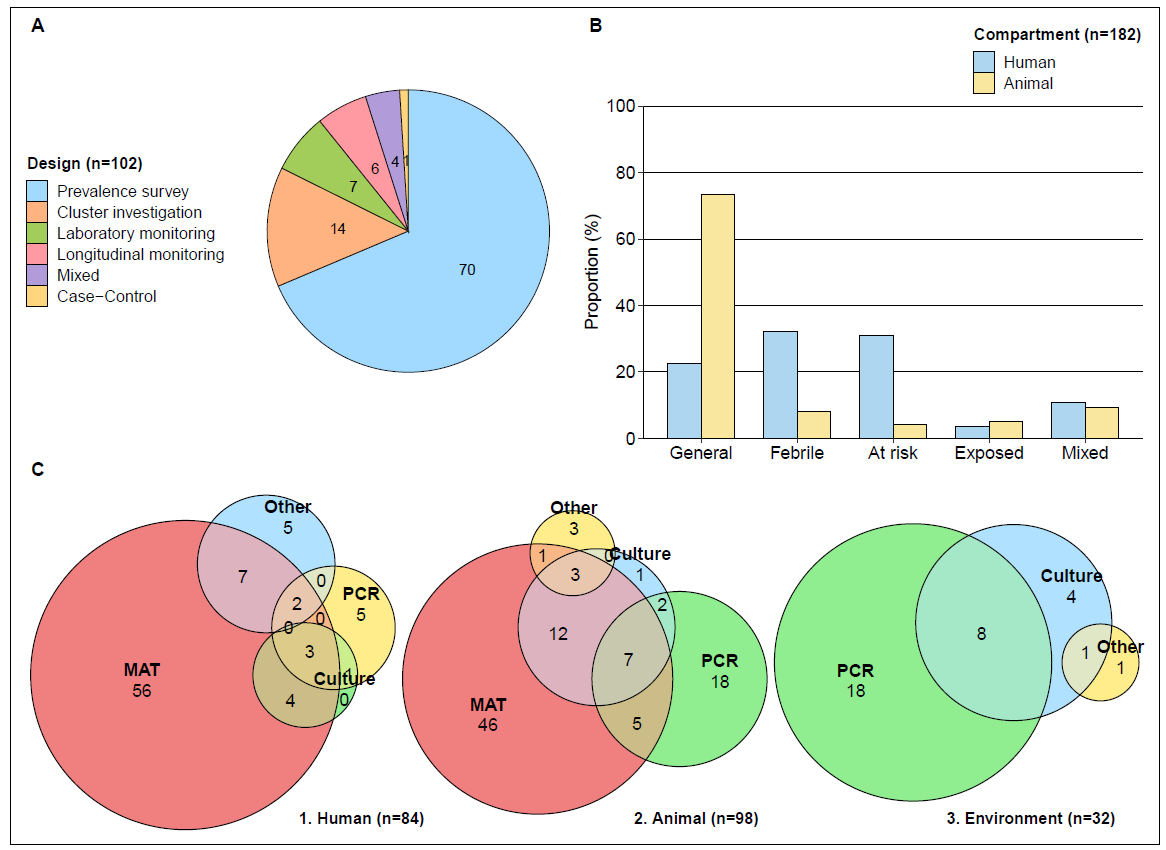


## Figure S3: Description of studies included. (A) Design distribution. (B) Proportions of types of study populations for humans and animal studies. (C) Number of studies using each test or combination of tests in (1) human, (2) animal and (3) environmental sampling.

## One study in human (MAT+Culture+Other) does not appear.

# Supplementary Material 6: Biospecimen.

## Table S4: Biospecimen used for human and animal tests.

|  | DF microscopy | Culture | PCR |
| --- | --- | --- | --- |
| Human | Urine 1/1 | Blood 3/10  Urine 5/10  Blood+Urine 2/10 | Blood 6/11  Urine 2/11  Feces 1/11  Serum+Urine 1/11  NA 1/11 |
| Animals without rodents, bandicoots and shrews | Urine 2/2 | Urine 15/20  Kidney 2/20  Blood+Urine 1/20  Blood+Urine+Kidney 1/20  Blood+Milk 1/20 | Urine 15/27  Blood 2/27  Kidney 4/27  Feces 1/27  Mucus 1/27  Blood+Urine 2/27  Urine+Kidney 1/27  Blood+Urine+Kidney 1/27 |
| Rodents, bandicoots and shrews | Urine 1/2  Kidney 1/2 | Kidney 6/12  Urine 2/12  Blood 1/12  Urine+Kidney 1/12  Kidney+Liver 1/12  Blood+Urine+Kidney 1/12 | Urine 3/23  Kidney 14/23  Spleen 1/23  Urine+Kidney 1/23  Kidney+Liver 2/23  Spleen+Kidney 1/23  Blood+Urine+Kidney 1/23 |

# Supplementary Material 7: Searching for links between One Health compartments.

## Table S5: Statistical methods looking for links between One Health components.

| Article | Link | | | Statistical method | Variable |
| --- | --- | --- | --- | --- | --- |
|  | H-A | H-E | A-E |  |  |
| Campagnolo et al (2000) | 1 | 0 | 0 | Odds Ratio | Animal urine splashed on face |
| Vanasco et al (2000) | 1 | 1 | 0 | χ² test or Fisher test, Yates correction or Pearson correlation | Dog contact, rodent exposure, animal contact, contact with flood water, exposure with rainwater |
| Ramakrishnan et al (2003) | 1 | 1 | 1 | χ² test, Fisher test | Animal contact, kind of drinking water |
| Ren et al (2005) | 0 | 1 | 0 | χ² test | Before/after flooding |
| Langoni et al (2008) | 1 | 1 | 1 | χ² test, Fisher test | Animal: kind of soil, drinking water, rodent exposure, Human: kind of soil, drinking water, rodent exposure, milk consumption |
| Zhou et al (2009) | 0 | 1 | 1 | χ² test | Floods occurrence and proximity |
| Jorge Da Silva et al (2015) | 0 | 0 | 1 | Fisher test | Positive rate according to the biome |
| Lugo-Chavez et al (2015) | 1 | 0 | 0 | χ² test | Owning a pet |
| Barragan et al (2016) | 0 | 1 | 0 | Fisher test with post-hoc, Odds Ratio | Rainfall |
| Parveen et al (2016) | 1 | 1 | 0 | Odds Ratio: matched analysis | Animal contact, river swimming, rat infestation, proximity to water bodies |
| Biscornet et al (2017) | 0 | 0 | 1 | Multivariate logistic regression | Urban/rural, Dry/rainy season |
| Jorge et al (2017) | 1 | 1 | 1 | Linear regression analyses, Pearson's correlation | Precipitations, human seroprevalence, canine, bovine, equine seroprevalence |
| Habus et al (2017) | 0 | 1 | 0 | Poisson distribution, logarithm link | Weather situation |
| Chadsuthi et al (2017) | 1 | 0 | 0 | Spearman correlation | Human/animal seroprevalences |
| Shrestha et al (2018) | 1 | 1 | 0 | Multivariate logistic regression | Dog exposure, contact with animal urine or feces, rice field work after rainfall, animal exposure, rodent exposure, work on bovine farm, drinking water |
| Salmon-Mulanovich (2019) | 1 | 0 | 0 | Multivariate logistic regression | Contact with backyard birds, owning any animal |
| Meny et al (2019) | 1 | 1 | 0 | Multivariate logistic regression | Animal contact, rodent exposure, flood exposure, usage of unsafe water |
| Calderon et al (2019) | 1 | 1 | 0 | Bivariate logistic regression | Contact with horse fluids, owning a dog, recent swimming |
| Goh et al (2019) | 1 | 1 | 0 | Multivariate logistic regression | Animal contact, rodent exposure, time in contact with dog, flood at home, monsoon |
| Mendoza Sanchez et al (2020) | 1 | 0 | 0 | χ² test | Animal contact |
| Murcia et al (2020) | 1 | 1 | 0 | Bivariate logistic regression | Humans: contact with dog fluid, owning a dog, recent swimming, Animal: drinking water, water management, rainwater management, pipes, septic tank access |
| Grimm et al (2020) | 1 | 0 | 1 | Multivariate logistic regression, Poisson regression | Correlation between animal seroprevalence and environmental positivity rate, Animal : human/forest proximity |
| do Nascimiento Benitez et al (2021) | 1 | 1 | 0 | Multivariate logistic regression | House with positive dogs, rat exposure, dirty backyard, nearby forest |
| Mgode et al (2021) | 0 | 1 | 1 | χ² test | According to the biome |
| Machado et al (2021) | 0 | 0 | 1 | Fisher test | According to the kind of environment: natural area or agricultural and anthropized area |
| Msemwa et al (2021) | 1 | 1 | 0 | Multivariate logistic regression | Rodent exposure, animal vaccination, nearby rice field, fallow land near the house |
| Setyaningsih et al (2022) | 1 | 1 | 0 | OR: relative odds | River presence, rat exposure, nearby rice field, owning a pet, water activity, field activity |
| Cunha et al (2022) | 1 | 1 | 0 | OR: relative odds, χ² test | Cat exposure, rat exposure, exposure to rat feces, history of flooding, presence of food remains |
| Richard et al (2022) | 0 | 0 | 1 | Spearman’s rank correlation, Shapiro-Wilk test | Correlation between rainfall and leptospires concentration |

## Table S6: Risk factors identified using a statistical approach by the studies.

|  |  | N studies |  |
| --- | --- | --- | --- |
| Humans | Presence of animal contacts | 11 | Benitez et al., 2020; Goh et al., 2019; Lugo-Chávez et al., 2015; Meny et al., 2022, 2019; Ochoa et al., 2000; Rodriguez et al., 2020; Salmon-Mulanovich et al., 2019; Sanhueza et al., 2017; Setyaningsih et al., 2022; Shrestha et al., 2018 |
|  | Occupation | 8 | Dreyfus et al., 2021; Meny et al., 2022, 2019; Natarajaseenivasan et al., 2002; Ochoa et al., 2000; Rodriguez et al., 2020; Setyaningsih et al., 2022; Shrestha et al., 2018 |
|  | Increased rainfall, flooding, humidity | 7 | Goh et al., 2019; Habus et al., 2017; Meny et al., 2019; Ren et al., 2005; Richard et al., 2022; Shrestha et al., 2018; Vanasco et al., 2000) |
|  | At-risk practice | 5 | Calderón et al., 2019; Campagnolo et al., 2000; Dreyfus et al., 2021; Parveen et al., 2016; Setyaningsih et al., 2022 |
|  | Lack of hygiene | 5 | Campagnolo et al., 2000; Langoni et al., 2008; Meny et al., 2019; Parveen et al., 2016; Shrestha et al., 2018 |
|  | Age | 3 | Mgode et al., 2019; Ochoa et al., 2000; Roqueplo et al., 2019 |
|  | Presence of rodents | 3 | Goh et al., 2019; Meny et al., 2019; Parveen et al., 2016 |
|  | Male | 2 | Shrestha et al., 2018; Van et al., 2017 |
|  | Young | 2 | Meny et al., 2019; Salmon-Mulanovich et al., 2019 |
|  | Proximity to water | 1 | Parveen et al., 2016 |
|  | Low level of education | 1 | Msemwa et al., 2021 |
|  | Canine seroprevalence | 1 | Jorge et al., 2017 |
|  | High temperature | 1 | Calderón et al., 2019 |
|  | Non-urban area | 1 | Goh et al., 2019 |
|  | Proximity to forest | 1 | Benitez et al., 2020 |
|  | Clinic signs | 1 | Meny et al., 2019 |
|  | Living in slum | 1 | Meny et al., 2019 |
|  | Other | 2 | Sanhueza et al., 2017, Van et al., 2017 |
| Animals | Presence of other animals | 5 | Benitez et al., 2020; Cunha et al., 2022; Goh et al., 2019; Murcia et al., 2020; Ochoa et al., 2000 |
|  | Lack of hygiene | 5 | Benitez et al., 2020; Calderón et al., 2019; Langoni et al., 2008; Murcia et al., 2020; Ochoa et al., 2000 |
|  | Increased of flooding, precipitation level | 4 | Biscornet et al., 2017; Cortez et al., 2018; Cunha et al., 2022; Jorge et al., 2017 |
|  | Presence of rodents | 3 | Biscornet et al., 2017; Goh et al., 2019; Ochoa et al., 2000 |
|  | Proximity to forest | 2 | Benitez et al., 2020; Machado et al., 2021 |
|  | Male | 2 | Benitez et al., 2020; Grimm et al., 2020 |
|  | Age | 2 | Grimm et al., 2020; Murcia et al., 2020 |
|  | Urban area | 2 | Biscornet et al., 2017; Goh et al., 2019 |
|  | Human seroprevalence | 1 | Jorge et al., 2017 |
|  | Animal seroprevalence | 1 | Jorge et al., 2017 |
|  | High temperature | 1 | Calderón et al., 2019 |
|  | At-risk practice | 1 | Calderón et al., 2019 |

# Supplementary Material 8: Risk of bias assessment results.


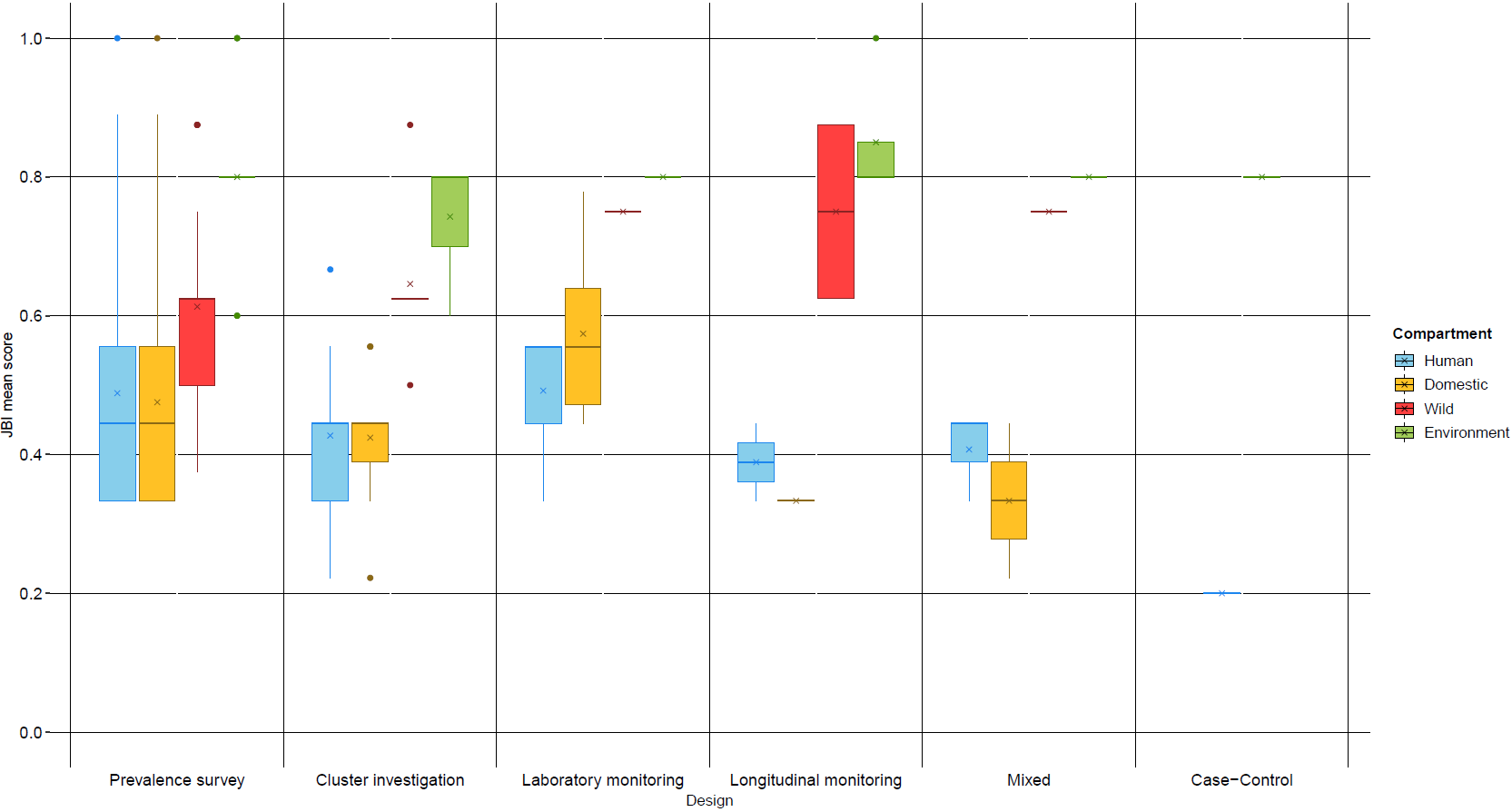


## Figure S4: Mean score by study design and compartment.

Most components (239/248) were considered to have an appropriate sample frame to address the target population (Q1), to use valid methods to measure the condition (246/248) (Q6) and to measure the condition in a standardized and reliable way among participants (246/248) (Q7). A minority of human components (12/84, 14·3%) and domestic animal component (17/76, 22·4%) satisfied the criteria on subject recruitment (Q2) and on adequate sample size (Q3) 14/84 (16·6%) for human component, 13/76 (17.1%) for domestic animal component and 7/56 (12·5%) for wild animal component. Concerning the description of subject and settings (Q4), 44/84 (52·4%) validated for human component, 18/76 (23·7%) for domestic animal component, 33/56 (58·9%) for wild animal component and 27/32 (84·4%) for environmental component. The criteria on coverage of the identified sample and existence of a selection bias (Q5) was not satisfied by most components: 9/84 human component, 10/76 domestic animal component and 18/56 wild animal component. As well as appropriate statistical analysis (Q8): 26/84 (30·9%) human component, 22/76 (28·9%) domestic animal component, 14/56 (25·0%) wild animal component and 4/32 (12·5%) environmental component. Concerning the management of the response rate (Q9), 9/84 human component satisfied this criteria and 9/76 domestic animals component.

# Supplementary Material 9: Meta-regressions with human seroprevalence outcome.

## Table S7a: Meta-regression with mixed-effects model, effect of the type of study population on human seroprevalence.

| Variable | Estimate | | P-value | |
| --- | --- | --- | --- | --- |
|  | β | 95% IC |  |  |
| Febrile | 0.01 | [-0.91 ; 0.92] | 0.9875 |  |
| At risk | -0.29 | [-1.20 ; 0.62] | 0.5288 |  |
| Exposed | 1.79 | [0.02 ; 3.56] | 0.0473 | * |
| Mixed | 0.99 | [-0.22 ; 2.20] | 0.1082 |  |

Reference: General population

I² = 98.38%

## Table S7b: Meta-regression with mixed-effects model, effect of type of risk the on human at risk seroprevalence.

| Variable | Estimate | | P-value |
| --- | --- | --- | --- |
|  | β | [95% IC] |  |
| Farmer | 0.16 | [-1.44 ; 1.76] | 0.8423 |
| Meat industry | -1.88 | [-4.08 ; 0.33] | 0.0954 |
| Security | -2.01 | [-4.52 ; 0.50] | 0.1167 |
| Miner | 1.12 | [-2.01 ; 4.26] | 0.4822 |
| Multi work | -0.20 | [-2.55 ; 2.14] | 0.8655 |
| Practice | -20.95 | [-2027.96 ; 1986.05] | 0.9837 |

Reference: Animal care

I² = 95.33%

## Table S7c: Meta-regression with mixed-effects model, effect of the region on human seroprevalence, adjusted on study population type.

| Variable | Estimate | | P-value | |
| --- | --- | --- | --- | --- |
|  | β | 95% IC |  |  |
| North America | 2.06 | [0.26 ; 3.86] | 0.0251 | * |
| South America | 0.57 | [-0.38 ; 1.51] | 0.2386 |  |
| West Asia | 1.10 | [0.22 ; 2.17] | 0.0455 | * |
| East Asia Pacific | 1.40 | [0.22 ; 2.58] | 0.0198 | * |
| Europe | 0.70 | [-0.50 ; 1.90] | 0.2537 |  |

Reference: Africa

I² = 98.61%

## Table S7d: Meta-regression with mixed-effects model, effect of the country on human seroprevalence, adjusted on study population type.

| Variable | Estimate | | P-value | |
| --- | --- | --- | --- | --- |
|  | β | 95% IC |  |  |
| Argentina | 0.85 | [-1.82 ; 3.52] | 0.5316 |  |
| Belize | 0.10 | [-2.48 ; 2.67] | 0.9418 |  |
| Bhutan | -1.64 | [-4.04 ; 0.76] | 0.1800 |  |
| Bolivia | -0.35 | [-2.84 ; 2.14] | 0.7802 |  |
| Cape Verde | -1.19 | [-3.78 ; 1.39] | 0.3654 |  |
| China | 0.96 | [-0.82 ; 2.75] | 0.2904 |  |
| Colombia | 1.33 | [0.27 ; 2.39] | 0.0140 | * |
| Croatia | 1.34 | [-0.49 ; 3.17] | 0.1506 |  |
| Egypt | 0.18 | [-1.51 ; 1.88] | 0.8320 |  |
| India | 1.53 | [0.34 ; 2.72] | 0.0116 | * |
| Iran | -0.92 | [-2.86 ; 1.01] | 0.3506 |  |
| Italia | 0.57 | [-1.28 ; 2.41] | 0.5474 |  |
| Madagascar | -2.03 | [-5.15 ; 1.10] | 0.2035 |  |
| Malaysia | 1.82 | [0.40 ; 3.25] | 0.0121 | * |
| Mexico | 1.69 | [-1.30 ; 4.69] | 0.2681 |  |
| Nepal | -0.21 | [-2.80 ; 1.92] | 0.8680 |  |
| New-Zealand | 0.21 | [-2.18 ; 2.60] | 0.8650 |  |
| Peru | 0.39 | [-1.95 ; 2.73] | 0.7447 |  |
| Philippines | 1.11 | [-1.34 ; 3.57] | 0.3742 |  |
| Senegal | 0.02 | [-2.34 ; 2.38] | 0.9866 |  |
| Seychelles | 0.28 | [-2.13 ; 2.69] | 0.8215 |  |
| Slovakia | 0.07 | [-1.47 ; 1.62] | 0.9254 |  |
| Taiwan | 0.77 | [-1.61 ; 3.14] | 0.5273 |  |
| Tanzania | 0.44 | [-0.81 ; 1.73] | 0.4995 |  |
| Thailand | 2.47 | [0.74 ; 4.21] | 0.0052 | ** |
| Ukraine | 0.97 | [-0.85 ; 2.78] | 0.2972 |  |
| Uruguay | 0.37 | [-1.15 ; 1.92] | 0.6215 |  |
| U.S.A. | 2.22 | [0.42 ; 4.03] | 0.0156 | * |
| U.S Virgin Islands | -1.12 | [-3.91 ; 1.67] | 0.4311 |  |

Reference: Brazil

I² = 97.31%

# Supplementary Material 10: Meta-regressions with animal seroprevalence outcome.

## Table S8a: Meta-regression with mixed-effects model, effect of the type of study population on animal seroprevalence.

| Variable | Estimate | | P-value | |
| --- | --- | --- | --- | --- |
|  | β | 95% IC |  |  |
| Febrile | -1.45 | [-2.26 ; -0.65] | 0.0004 | *** |
| At risk | 0.52 | [-1.20 ; 2.34] | 0.5571 |  |
| Exposed | 0.09 | [-1.09 ; 1.27] | 0.8837 |  |
| Mix | -0.55 | [-1.18 ; 0.07] | 0.0832 |  |

Reference: General population

I² = 99.88%

## Table S8b: Meta-regression with mixed-effects model, effect of the region on animal seroprevalence, adjusted on study population type.

| Variable | Estimate | | P-value | |
| --- | --- | --- | --- | --- |
|  | β | 95% IC |  |  |
| North America | 1.78 | [0.62 ; 2.94] | 0.0025 | ** |
| South America | 1.88 | [1.13 ; 2.63] | <0.0001 | *** |
| West Asia | 1.36 | [0.39 ; 2.32] | 0.0060 | ** |
| East Asia Pacific | 1.37 | [0.56 ; 2.19] | 0.0010 | *** |
| Africa | 1.00 | [0.18 ; 1.81] | 0.0164 | * |

Reference: Europe

I² = 99.86%

## Table S8c: Meta-regression with mixed-effects model, effect of the country on animal seroprevalence, adjusted on study population type.

| Variable | Estimate | | P-value | |
| --- | --- | --- | --- | --- |
|  | β | 95% IC |  |  |
| Argentina | 3.49 | [-0.03 ; 6.96] | 0.0481 | * |
| Belize | 2.21 | [0.53 ; 3.89] | 0.0100 | * |
| Bhutan | 0.66 | [-1.21 ; 2.53] | 0.4896 |  |
| Bolivia | 0.05 | [-1.35 ; 1.45] | 0.9442 |  |
| Cape Verde | -3.86 | [-5.19 ; -2.54] | <0.0001 | *** |
| China | -2.17 | [-4.81 ; 0.48] | 0.1082 |  |
| Colombia | 0.69 | [-0.17 ; 1.55] | 0.1169 |  |
| Croatia | -1.31 | [-2.57 ; -0.05] | 0.0420 | * |
| Egypt | 0.34 | [-0.55 ; 1.22] | 0.4603 |  |
| France | 0.76 | [-1.81 ; 3.32] | 0.5640 |  |
| India | 0.47 | [-0.42 ; 1.36] | 0.3031 |  |
| Iran | -1.53 | [-2.79 ; -0.27] | 0.0173 | * |
| Italia | -1.82 | [-3.60 ; -0.04] | 0.0452 | * |
| Malaysia | -0.26 | [-1.43 ; 0.91] | 0.6617 |  |
| Mexico | 0.46 | [-0.93 ; 1.84] | 0.5186 |  |
| Nepal | 1.37 | [-0.76 ; 3.50] | 0.2087 |  |
| New-Zealand | 0.02 | [-1.52 ; 1.56] | 0.9797 |  |
| Nicaragua | 0.45 | [-2.59 ; 3.49] | 0.7712 |  |
| Peru | 0.86 | [-0.60 ; 2.31] | 0.2501 |  |
| Philippines | 0.73 | [-1.25 ; 2.71] | 0.4701 |  |
| Poland | -1.00 | [-3.04 ; 1.03] | 0.3347 |  |
| Senegal | 1.01 | [-0.30 ; 2.32] | 0.1275 |  |
| Slovakia | -1.35 | [-2.55 ; -0.14] | 0.0293 | * |
| Taiwan | -1.54 | [-2.96 ; -0.13] | 0.0329 | * |
| Tanzania | -0.52 | [-1.40 ; 0.36] | 0.2477 |  |
| Thailand | 0.41 | [-1.41 ; 2.24] | 0.6565 |  |
| Ukraine | -1.15 | [-2.89 ; 0.58] | 0.1927 |  |
| Uruguay | 0.59 | [-1.00 ; 2.17] | 0.4676 |  |
| U.S.A. | 0.32 | [-0.85 ; 1.49] | 0.5887 |  |
| U. S. Virgin Islands | 15.93 | [-693.65 ; 725.52] | 0.9649 |  |

Reference: Brazil

I² = 98.83%

## Table S8d: Meta-regression with mixed-effects model, effect of the animal type on animal seroprevalence, adjusted on study population type.

| Variable | Estimate | | P-value |
| --- | --- | --- | --- |
|  | β | [95% IC] |  |
| Wild | -0.53 | [-1.07 ; 0.02] | 0.0580 |

Reference: Domestic

I² = 99.88%

## Table S8e: Meta-regression with mixed-effects model, effect of the animal type on domestic animal seroprevalence, adjusted on study population type.

| Variable | Estimate | | P-value |
| --- | --- | --- | --- |
|  | β | [95% IC] |  |
| Pet | 0.28 | [-0.31 ; 0.87] | 0.3537 |

Reference: Livestock

I² = 99.78%

## Table S8f: Meta-regression with mixed-effects model, effect of the animal type on wild animal seroprevalence, adjusted on study population type.

| Variable | Estimate | | P-value |
| --- | --- | --- | --- |
|  | β | [95% IC] |  |
| Rodent | -0.62 | [-1.63 ; 0.40] | 0.2324 |

Reference: Wild non-rodent

I² = 99.37%

## Table S8g: Meta-regression with mixed-effects model, effect of the specie on animal seroprevalence, adjusted on study population type.

| Variable | Estimate | | P-value | |
| --- | --- | --- | --- | --- |
|  | β | 95% IC |  |  |
| Bovine | 1.05 | [0.23 ; 1.86] | 0.0116 | * |
| Camel | 0.37 | [-2.08 ; 2.82] | 0.7687 |  |
| Cat | 0.03 | [-1.40 ; 1.46] | 0.9673 |  |
| Dog | 1.14 | [0.34 ; 1.93] | 0.0051 | ** |
| Equine | 1.43 | [0.43 ; 2.42] | 0.0049 | ** |
| Pig | 0.48 | [-0.43 ; 1.40] | 0.3011 |  |
| Poultry | 3.32 | [0.20 ; 6.45] | 0.0370 | * |
| Small ruminants | -0.03 | [-0.88 ; 0.82] | 0.9504 |  |
| Bandicoot | 1.81 | [-0.51 ; 4.13] | 0.1260 |  |
| Bat | -20.15 | [-50,732 ; 50,691] | 0.9994 |  |
| Bird | 0.64 | [-2.39 ; 3.68] | 0.6777 |  |
| Boar | 0.27 | [-1.81 ; 2.35] | 0.7983 |  |
| Civet | 1.81 | [-2.28 ; 5.91] | 0.3848 |  |
| Coati | 2.66 | [-1.21 ; 6.53] | 0.1772 |  |
| Deer | -1.35 | [-3.60 ; 0.88] | 0.2385 |  |
| Feral cat | -19.52 | [-18,932.69 ; 18,893.64] | 0.9984 |  |
| Fish | -0.80 | [-4.15 ; 2.55] | 0.6386 |  |
| Fox | 2.15 | [0.13 ; 4.17] | 0.0373 | * |
| Lion | 1.83 | [-2.26 ; 5.92] | 0.3812 |  |
| Marmot | -20.07 | [-18,364.98 ; 18,324.85] | 0.9983 |  |
| Monkey | 2.03 | [0.17 ; 3.89] | 0.0326 | * |
| Opossum | 2.57 | [-0.12 ; 5.27] | 0.0611 |  |
| Rabbit | -17.51 | [-40,517.95 ; 40,482.94] | 0.9993 |  |
| Racoon | 2.01 | [-1.01 ; 5.04] | 0.1918 |  |
| Shrew | -0.76 | [-2.68 ; 1.16] | 0.4383 |  |
| Snake | -1.05 | [-1.69 ; 3.80] | 0.4517 |  |
| Tatou | 0.97 | [-1.72 ; 3.66] | 0.4811 |  |
| Wolf | -16.11 | [-13,868.43 ; 13,836.21] | 0.9982 |  |
| Zebra | -18.38 | [-33,754.80 ; 33,718.05] | 0.9991 |  |

Reference: Rodent

I² = 98.31%

# Supplementary Material 11: Meta-regressions with environmental positive rate outcome.

## Table S9a: Meta-regression with mixed-effects model, effect of the nature of sampling on environmental positive rate.

| Variable | Estimate | | P-value |
| --- | --- | --- | --- |
|  | β | [95% IC] |  |
| Water | -0.86 | [-2.09 ; 0.36] | 0.1654 |

Reference: Soil

I² = 96.16%

## Table S9b: Meta-regression with mixed-effects model, effect of the region on environmental positive rate.

| Variable | Estimate | | P-value | | | | |
| --- | --- | --- | --- | --- | --- | --- | --- |
|  | β | 95% IC |  | | | | |
| North America | -2.00 | [-3.92 ; -0.08] | 0.0794 | | | | * |
| West Asia | -0.83 | [-2.59 ; 0.93] | 0.3563 | | |  | |
| East Asia | -0.88 | [-2.05 ; 0.29] | 0.1416 | |  | | |
| Africa | -0.33 | [-3.26 ; 2.60] | 0.8275 | |  | | |
| Europe | -1.92 | [-3.62 ; -0.21] | 0.0274 | * | | | |

Reference: East Asia

I² = 87.31%

# Supplementary Material 12: Meta-regression models seeking for links between compartments.

## Table S10a: Meta-regression analyses seeking for associations between human and animal seroprevalences.

| Outcome | Tested association | N | Association type | Estimate | | P-value | |
| --- | --- | --- | --- | --- | --- | --- | --- |
|  |  |  |  | β | 95% CI |  | |
| Human | Animal | 65 | Positive | 2.14 | [0.42 ; 3.87] | 0.0150 | * |
|  | Domestic | 57 | Positive | 2.37 | [0.60 ; 4.13] | 0.0087 | ** |
|  | Pet | 36 | - | 2.67 | [-0.16 ; 5.50] | 0.0647 |  |
|  | Dog | 34 | - | 2.19 | [-0.98 ; 5.37] | 0.1755 |  |
|  | Livestock | 40 | Positive | 2.05 | [0.16 ; 3.93] | 0.0335 | * |
|  | Bovine | 27 | Positive | 2.56 | [0.34 ; 4.77] | 0.0238 | * |
|  | Small ruminants | 20 | Positive | 2.19 | [0.06 ; 4.32] | 0.0436 | * |
|  | Pig | 17 | - | 1.33 | [-1.79 ; 4.46] | 0.4033 |  |
|  | Equine | 15 | - | -1.53 | [-3.63 ; 0.57] | 0.1529 |  |
|  | Wild | 32 | - | 2.43 | [-0.76 ; 5.62] | 0.1349 |  |
|  | Rodent | 23 | - | 1.20 | [-2.44 ; 4.85] | 0.5172 |  |
|  | Wild non-rodent | 14 | Positive | 4.03 | [1.45 ; 6.60] | 0.0022 | ** |
| Animal | Human | 205 | Positive | 2.90 | [1.62 ; 4.19] | <0.0001 | *** |
| Domestic |  | 140 | Positive | 2.88 | [1.45 ; 4.30] | <0.0001 | *** |
| Pet |  | 45 | - | 1.56 | [-0.31 ; 3.43] | 0.1011 |  |
| Dog |  | 40 | - | 1.40 | [-0.61 ; 3.41] | 0.1733 |  |
| Livestock |  | 95 | Positive | 3.28 | [1.36 ; 5.20] | 0.0008 | *** |
| Bovine |  | 30 | Positive | 3.50 | [0.90 ; 6.10] | 0.0084 | ** |
| Small ruminants |  | 29 | Positive | 5.85 | [1.58 ; 10.13] | 0.0073 | ** |
| Pig |  | 17 | Positive | 4.25 | [0.63 ; 7.87] | 0.0214 | * |
| Equine |  | 15 | - | -1.96 | [-6.27 ; 2.34] | 0.3715 |  |
| Wild |  | 65 | - | 2.32 | [-0.27 ; 4.91] | 0.0793 |  |
| Rodent |  | 26 | - | 1.60 | [-1.63 ; 4.82] | 0.3319 |  |
| Wild non-rodent |  | 37 | Positive | 4.79 | [1.43 ; 8.16] | 0.0053 | ** |

## Table S10b: Meta-regression analyses seeking for associations between the environment positivity rate and animal and human seroprevalences.

| Outcome | Tested association | | | N | Association type | | | Estimate | | | P-value | | | | | | |
| --- | --- | --- | --- | --- | --- | --- | --- | --- | --- | --- | --- | --- | --- | --- | --- | --- | --- |
|  |  | | |  |  | | | β | 95% CI | |  | | | | | | |
| Animal | | Environment | 22 | | | - | 2.90 | | | [-1.08 ; 6.87] | | 0.1534 | | | | |  |
| Domestic | |  | 19 | | | Positive | 3.28 | | | [0.10 ; 6.47] | | 0.0435 | | | | * | |
| Livestock | |  | 13 | | | Positive | 3.92 | | | [0.56 ; 7.28] | | 0.0224 | | | * | | |
| Environment | | Animal | 11 | | | - | 1.97 | | | [-3.09 ; 7.02] | | 0.4460 | |  | | | |
|  | | Human | 13 | | | - | 0.67 | | | [-2.65 ; 3.99] | | 0.6930 |  | | | | |

## Table S10c: Meta-regression analyses seeking for associations between seroprevalences of different animal categories.

| Outcome | Tested association | N | Association type | Estimate | | P-value | |
| --- | --- | --- | --- | --- | --- | --- | --- |
|  |  |  |  | β | 95% CI |  | |
| Domestic | Wild | 78 | Positive | 1.67 | [0.11 ; 3.23] | 0.0363 | * |
|  | Rodent | 49 | - | 1.38 | [-1.26 ; 4.02] | 0.3060 |  |
|  | Wild non-rodent | 24 | - | 0.83 | [-1.36 ; 3.03] | 0.4568 |  |
| Pet | Livestock | 26 | - | 2.53 | [-0.07 ; 5.13] | 0.0562 |  |
|  | Wild | 22 | - | 2.25 | [-0.02 ; 4.51] | 0.0516 |  |
|  | Rodent | 16 | - | 2.38 | [-0.97 ; 5.74] | 0.1636 |  |
| Dog | Livestock | 23 | Positive | 3.43 | [0.69 ; 6.17] | 0.0142 | * |
|  | Bovine | 18 | - | 2.57 | [-0.50 ; 5.64] | 0.1011 |  |
|  | Small ruminants | 14 | Positive | 7.58 | [2.34 ; 12.82] | 0.0046 | ** |
|  | Pig | 10 | Positive | 6.29 | [1.70 ; 10.87] | 0.0072 | ** |
|  | Wild | 20 | - | 2.31 | [-0.13 ; 4.75] | 0.0634 |  |
|  | Rodent | 13 | - | 1.79 | [-2.13 ; 5.71] | 0.3714 |  |
| Livestock | Dog | 51 | Positive | 2.91 | [1.10 ; 4.72] | 0.0016 | ** |
|  | Wild | 56 | - | 1.50 | [-0.40 ; 3.40] | 0.1210 |  |
|  | Rodent | 33 | - | 1.41 | [-2.23 ; 5.06] | 0.4471 |  |
|  | Wild non-rodent | 19 | - | 0.56 | [-2.00 ; 3.12] | 0.6659 |  |
| Bovine | Dog | 15 | - | 2.36 | [-0.98 ; 5.70] | 0.1656 |  |
|  | Small ruminants | 18 | Positive | 5.34 | [2.06 ; 8.63] | 0.0014 | ** |
|  | Pig | 15 | - | 1.74 | [-2.63 ; 6.12] | 0.4349 |  |
|  | Wild | 18 | - | 2.34 | [-0.32 ; 4.99] | 0.0843 |  |
|  | Rodent | 13 | Positive | 3.47 | [0.27 ; 6.68] | 0.0335 | * |
| Small ruminants | Dog | 16 | Positive | 3.04 | [0.68 ; 5.40] | 0.0117 | * |
|  | Bovine | 21 | Positive | 4.47 | [2.61 ; 6.34] | <0.0001 | *** |
|  | Pig | 13 | Positive | 7.16 | [4.09 ; 10.24] | <0.0001 | *** |
|  | Wild | 19 | - | 2.86 | [-0.06 ; 5.78] | 0.0550 |  |
|  | Rodent | 10 | - | -0.11 | [-6.65 ; 6.43] | 0.9744 |  |
| Pig | Bovine | 13 | - | 2.08 | [-1.40 ; 5.57] | 0.2420 |  |
|  | Small ruminants | 10 | Positive | 5.81 | [0.99 ; 10.64] | 0.0183 | * |
| Equine | Dog | 10 | - | 1.56 | [-1.62 ; 4.74] | 0.3369 |  |
|  | Small ruminants | 11 | - | 0.45 | [-4.94 ; 5.85] | 0.8687 |  |
| Wild | Domestic | 50 | - | 4.08 | [-0.07 ; 8.22] | 0.0537 |  |
|  | Dog | 37 | Positive | 6.96 | [1.03 ; 12.90] | 0.0214 | * |
|  | Livestock | 45 | - | 3.29 | [-1.47 ; 8.05] | 0.1753 |  |
|  | Bovine | 37 | - | 5.06 | [-0.18 ; 10.30] | 0.0586 |  |
|  | Small ruminants | 37 | Positive | 5.80 | [0.13 ; 11.47] | 0.0451 | * |
|  | Pig | 18 | - | -5.45 | [-28.87 ; 17.96] | 0.6480 |  |
| Rodent | Domestic | 22 | - | 2.57 | [-1.72 ; 6.87] | 0.2401 |  |
|  | Dog | 14 | Positive | 6.26 | [0.44 ; 12.08] | 0.0349 | * |
|  | Livestock | 20 | - | 0.94 | [-3.54 ; 5.42] | 0.6819 |  |
|  | Bovine | 15 | - | 4.60 | [-0.74 ; 9.94] | 0.0912 |  |
|  | Small ruminants | 12 | - | 4.58 | [-2.15 ; 11.30] | 0.1823 |  |
| Wild non-rodent | Domestic | 26 | - | 1.95 | [-4.15 ; 8.05] | 0.5316 |  |
|  | Dog | 21 | - | 5.77 | [-2.61 ; 14.15] | 0.1772 |  |
|  | Livestock | 24 | - | 2.11 | [-5.79 ; 10.01] | 0.6010 |  |
|  | Bovine | 21 | - | -1.75 | [-7.73 ; 4.22] | 0.5653 |  |
|  | Small ruminants | 24 | - | 7.13 | [-2.40 ; 16.66] | 0.1428 |  |
|  | Rodent | 11 | - | -5.54 | [-24.70 ; 13.62] | 0.5710 |  |

# Supplementary Material 13: Exploration of PCR data.

## Table S11: Meta-regression analyses of PCR results explained by various positivity rate.

| Outcome | Tested association | N | Association type | Estimate | | P-value | |
| --- | --- | --- | --- | --- | --- | --- | --- |
|  |  |  |  | β | 95% CI |  | |
| Animal | Human sero | 20 | Positive | 10.44 | [0.08 ; 20.79] | 0.0482 | * |
|  | Human PCR | 19 | - | 1.37 | [-2.28 ; 5.02] | 0.4624 |  |
|  | Environment | 32 | - | 2.38 | [-2.51 ; 7.27] | 0.3393 |  |
|  | Self-sero | 10 | Positive | 10.82 | [4.23 ; 17.41] | 0.0013 | ** |
| Domestic | Human sero | 12 | - | 24.43 | [-5.50 ; 54.36] | 0.1096 |  |
|  | Human PCR | 14 | - | 2.17 | [-10.30 ; 14.64] | 0.7327 |  |
|  | Environment | 14 | - | -10.01 | [-22.33 ; 2.30] | 0.1110 |  |
| Wild | Environment | 18 | - | 4.75 | [-0.20 ; 9.70] | 0.0602 |  |
| Environment | Animal PCR | 21 | - | 1.00 | [-2.49 ; 4.48] | 0.5754 |  |

sero = seroprevalence

# Supplementary Material 14: Exploration to validate the results of the meta-analysis.

## Table S12: Meta-regression analyses among subsets with a potentially better representativeness.

| Subset | N | Outcome | Tested association | Results | Previous results |
| --- | --- | --- | --- | --- | --- |
| Randomized studies: healthy population prevalence survey for humans and animals | 12 | Animal | Human | β=11.6  p=0.21 | Positive association |
|  | 11 | Domestic animal | Human | β=13.8  p=0.12 | Positive association |
| Healthy population with at least 100 samples for humans and animals | 66 | Animal | Human | β=3.2  p=0.08 | Positive association |
|  | 54 | Domestic | Human | β=1.04  p=0.58 | Positive association |
